# Postmenopausal hormone therapy and risk of stroke: A pooled analysis of data from population-based cohort studies

**DOI:** 10.1371/journal.pmed.1002445

**Published:** 2017-11-17

**Authors:** Germán D. Carrasquilla, Paolo Frumento, Anita Berglund, Christer Borgfeldt, Matteo Bottai, Chiara Chiavenna, Mats Eliasson, Gunnar Engström, Göran Hallmans, Jan-Håkan Jansson, Patrik K. Magnusson, Peter M. Nilsson, Nancy L. Pedersen, Alicja Wolk, Karin Leander

**Affiliations:** 1 Institute of Environmental Medicine, Karolinska Institutet, Stockholm, Sweden; 2 Department of Obstetrics and Gynecology, Skåne University Hospital, Lund University, Lund, Sweden; 3 Department of Public Health and Clinical Medicine, Sunderby Research Unit, Umeå University, Umeå, Sweden; 4 Department of Clinical Sciences, Lund University, Lund-Malmö, Sweden; 5 Department of Public Health and Clinical Medicine, Nutritional Research, Umeå University, Umeå, Sweden; 6 Department of Public Health and Clinical Medicine, Research Unit Skellefteå, Umeå University, Umeå, Sweden; 7 Department of Medical Epidemiology and Biostatistics, Karolinska Institutet, Stockholm, Sweden; Stanford University, UNITED STATES

## Abstract

**Background:**

Recent research indicates a favourable influence of postmenopausal hormone therapy (HT) if initiated early, but not late, on subclinical atherosclerosis. However, the clinical relevance of timing of HT initiation for hard end points such as stroke remains to be determined. Further, no previous research has considered the timing of initiation of HT in relation to haemorrhagic stroke risk. The importance of the route of administration, type, active ingredient, and duration of HT for stroke risk is also unclear. We aimed to assess the association between HT and risk of stroke, considering the timing of initiation, route of administration, type, active ingredient, and duration of HT.

**Methods and findings:**

Data on HT use reported by the participants in 5 population-based Swedish cohort studies, with baseline investigations performed during the period 1987–2002, were combined in this observational study. In total, 88,914 postmenopausal women who reported data on HT use and had no previous cardiovascular disease diagnosis were included. Incident events of stroke (ischaemic, haemorrhagic, or unspecified) and haemorrhagic stroke were identified from national population registers. Laplace regression was employed to assess crude and multivariable-adjusted associations between HT and stroke risk by estimating percentile differences (PDs) with 95% confidence intervals (CIs). The fifth and first PDs were calculated for stroke and haemorrhagic stroke, respectively. Crude models were adjusted for age at baseline only. The final adjusted models included age at baseline, level of education, smoking status, body mass index, level of physical activity, and age at menopause onset. Additional variables evaluated for potential confounding were type of menopause, parity, use of oral contraceptives, alcohol consumption, hypertension, dyslipidaemia, diabetes, family history of cardiovascular disease, and cohort. During a median follow-up of 14.3 years, 6,371 first-time stroke events were recorded; of these, 1,080 were haemorrhagic. Following multivariable adjustment, early initiation (<5 years since menopause onset) of HT was associated with a longer stroke-free period than never use (fifth PD, 1.00 years; 95% CI 0.42 to 1.57), but there was no significant extension to the time period free of haemorrhagic stroke (first PD, 1.52 years; 95% CI −0.32 to 3.37). When considering timing as a continuous variable, the stroke-free and the haemorrhagic stroke-free periods were maximal if HT was initiated approximately 0–5 years from the onset of menopause. If single conjugated equine oestrogen HT was used, late initiation of HT was associated with a shorter stroke-free (fifth PD, −4.41 years; 95% CI −7.14 to −1.68) and haemorrhagic stroke-free (first PD, −9.51 years; 95% CI −12.77 to −6.24) period than never use. Combined HT when initiated late was significantly associated with a shorter haemorrhagic stroke-free period (first PD, −1.97 years; 95% CI −3.81 to −0.13), but not with a shorter stroke-free period (fifth PD, −1.21 years; 95% CI −3.11 to 0.68) than never use. Given the observational nature of this study, the possibility of uncontrolled confounding cannot be excluded. Further, immortal time bias, also related to the observational design, cannot be ruled out.

**Conclusions:**

When initiated early in relation to menopause onset, HT was not associated with increased risk of incident stroke, regardless of the route of administration, type of HT, active ingredient, and duration. Generally, these findings held also for haemorrhagic stroke. Our results suggest that the initiation of HT 0–5 years after menopause onset, as compared to never use, is associated with a decreased risk of stroke and haemorrhagic stroke. Late initiation was associated with elevated risks of stroke and haemorrhagic stroke when conjugated equine oestrogen was used as single therapy. Late initiation of combined HT was associated with haemorrhagic stroke risk.

## Abstract 2

In a pooled analysis of data from 3 cohort studies, Karin Leander and colleagues examine the association between postmenopausal hormone therapy and risk of stroke.

## Author summary

**Why was this study done?:**

- Postmenopausal hormone therapy (HT) is the most effective treatment for menopausal symptoms but has been found to increase the risk of cardiovascular events in randomized controlled trials.
- Results from a recent randomized clinical trial showed that HT, if given within 6 years since menopause onset, significantly reduced atherosclerosis. However, studies evaluating the risk of hard clinical cardiovascular end points including stroke have shown discrepant results.
- The importance of timing of HT initiation and its route of administration, type, active ingredient, and duration for stroke and haemorrhagic stroke risk is unclear.

**What did the researchers do and find?:**

- Through pooling of data from 5 Swedish population-based cohort studies, we obtained a study sample of menopausal women that was suitable for conducting analyses to assess whether the number of stroke-free years was associated with the use of HT while considering several therapy-related factors: the timing of initiation in relation to menopause onset, route of administration, type, active ingredient, and duration of use. Stroke was studied as a composite end point including ischaemic, haemorrhagic, and unspecified stroke, although separate analyses of haemorrhagic stroke were also performed.
- HT, if initiated <5 years after menopause onset and regardless of regimen and duration, was associated with a reduced or null risk of future stroke. Findings for haemorrhagic stroke were generally corresponding.
- If single therapy was initiated late with regard to menopause onset and comprised conjugated equine oestrogens as the active ingredient, increased risks of stroke and haemorrhagic stroke were observed.

**What do these findings mean?:**

- This study provides support for the clinical relevance of timing of HT initiation for stroke risk.
- Despite the potential limitations inherent in an observational study design, these findings contribute to the scientific basis for informed clinical decisions about HT initiation for menopausal symptoms.
- The study results should not influence current practice that recommends against the use of HT for cardiovascular disease prevention.

## Introduction

Postmenopausal hormone therapy (HT) is recognized as the most effective treatment currently available for menopausal symptoms [1,2]. Nevertheless, findings of a number of observational studies and randomized controlled trials (RCTs) of the influence of HT on stroke risk have been inconsistent [3–9]. A systematic review based on 10 RCTs concluded that oral HT in postmenopausal women increases the risk of stroke [10]. However, subanalyses of this review concluded that there was no strong evidence on risk of stroke according to the timing of initiation of HT [10]. There are recent indications of a favourable influence on subclinical atherosclerosis from HT initiated early, but not late, in relation to onset of menopause [11]; however, the clinical relevance for hard end points such as stroke remains to be determined [12]. The importance of early initiation of HT in relation to stroke risk was addressed in a limited number of previous studies [9,13,14], and the findings were inconsistent. Further, few studies have assessed the influence of HT use on the risk of haemorrhagic stroke, and results have been inconclusive because of the limited number of cases and because timing of HT initiation was not considered [15–18]. In addition, the pathophysiology of haemorrhagic stroke is different from that of the more common ischaemic stroke [19]. Therefore, it seems important to address these conditions separately.

The importance of the route of administration, type, active ingredient, and duration of HT for stroke risk is unclear [10]. To our knowledge, no previous studies have compared the different routes of administration (oral, transdermal, or vaginal) and active ingredients (conjugated equine oestrogens [CEEs] or oestradiol) in relation to stroke risk. Observational studies and RCTs that compared combined (oestrogen and a progestin) and oestrogen-only HT in relation to stroke risk have reported inconsistent results [9,20,21]. The optimal duration of HT from the perspective of stroke risk also remains to be determined [10].

We aimed to assess the association between HT and the risk of developing stroke, considering haemorrhagic stroke separately, while taking into account the timing of initiation of HT in relation to the onset of menopause, route of administration, type of HT, active ingredient, and duration. Our analysis is based on combined data from a large number of postmenopausal women in 5 population-based Swedish cohorts.

## Methods

### Study design

The Regional Ethical Review Board in Stockholm approved the study, performed in accordance with the principles expressed in the Declaration of Helsinki. Written informed consent was obtained from all study participants. This study is based on the Combined cohorts of menopausal women—Studies of register-based health outcomes in relation to hormonal drugs (COMPREHEND) collaborative effort initiated in 2011. Five cohorts included in COMPREHEND and with available data (from a questionnaire or interviews) on menopausal status, age at menopause onset, use of HT, and age at HT initiation were invited to collaborate in the present study, and all agreed. The cohorts are described below. Baseline investigations were performed between 1987 and 2002, a period during which HT was frequently prescribed.

### Description of cohorts

The prospective, population-based Swedish Mammography Cohort (SMC) study was performed between 1 March 1987 and 14 December 1990. All women born during the period 1914–1948 and residing in Västmanland and Uppsala counties in central Sweden were invited to undergo a free mammography examination and complete a questionnaire to collect information about their height, weight, education, diet, and consumption of alcoholic beverages [22]. Of the 90,303 contacted women, a total of 66,651 (74%) completed the questionnaire. In September 1997, the 56,030 women who remained alive and resident in the study counties were sent a second questionnaire to update the original data and to collect further information about reproductive history, history of hypertension and diabetes mellitus and lifestyle factors (including smoking status); 39,227 (70%) of these women completed the second questionnaire [22].

The Northern Sweden Health and Disease Study (NSHDS) comprises 3 population-based subcohorts: the Mammary Screening Cohort (MSC), the Västerbotten Intervention Programme (VIP), and the Monitoring Trends and Determinants in Cardiovascular Disease (MONICA). The MSC was based on mammography screening (85% participation rate) and was initiated in 1995 for all women aged 50–69 years living in Västerbotten county. At screening (1995–2006), the women were also invited to participate in a study of several endemic diseases that included cardiovascular disease (CVD). Overall, 46% completed a questionnaire that was mainly concerning reproductive history; weight, height, and smoking status were also recorded. In the present study, the 27,708 participants in the MSC composed the core cohort, and 19,338 participants from the VIP and 1,053 from the MONICA subcohorts provided further data. A detailed description of the NHSDS has been reported [23].

In the Screening Across the Lifespan Twin (SALT), a substudy of the Swedish Twin Register, data were collected from 44,919 participants using computer-assisted telephone interviews. The SALT cohort consists of twin pairs (dizygotic and monozygotic) born up to 1958, identified from birth records. The interviews were conducted between 1998 and 2002 (73.6% participation rate). DNA analysis was used to determine zygosity for same-sex twins. The SALT study has been described previously [24].

The aim of the prospective, population-based Malmö Diet and Cancer study (MDCS) was to assess associations between cancer and lifestyle factors. The cohort comprises 17,035 women, 45–73 years of age, enrolled between 1991 and 1996 (born 1923–1950), with a participation rate of approximately 40%. Baseline examinations included anthropometric measures (e.g., body mass index), blood collection, a questionnaire to ascertain medications, sociodemographic characteristics, lifestyle, health status, and reproductive factors. Further details have been reported previously [25]. In contrast to the other cohorts, only a proportion (20%) of the women in the MDCS were asked to provide information that enabled the timing of HT initiation to be determined.

All women aged 50–59 years (born 1935–1945) and living in southern Sweden were invited to participate in a health survey as part of the Women’s Health in the Lund Area (WHILA). Between 2 December 1995 and 3 February 2000, a total of 6,916 participants (64% of the overall population of 10,766 women in 1995) underwent a physical examination and completed a questionnaire that included questions about health status, medications, sociodemographic characteristics, and alcohol consumption. Further details have been reported previously [26].

We excluded women with previous CVD, identified from the Swedish National Patient Register, as well as women who were not menopausal and those who were menopausal but reported an age at menopause outside the range of 40–59 years. After also excluding women for whom information on HT use was missing, a total of 88,914 were included in the study (S1 Fig) (i.e., 30,832, 20,108, 18,818, 13,700, and 5,456 from the SMC, NSHDS, SALT, MDCS, and WHILA cohorts, respectively).

### Follow-up and definition of end points

Utilizing the Swedish National Patient Register and the Swedish Cause of Death Register, incident cases of stroke (ischaemic, haemorrhagic, or unspecified; International Classification of Diseases [ICD] codes ICD-9 430–434/436–438 and ICD-10 I60–I69) and of haemorrhagic stroke specifically (ICD-9 430–432 and ICD-10 I60–I62) during the follow-up period were identified. In addition, main diagnoses and the underlying causes of death were recorded. The end of follow-up varied between cohorts, from 31 December 2010 to 31 December 2013.

### Classification of HT

Women who reported current or previous use of HT were categorized as ever users. Ever users who reported initiating HT within the previous 12 months were categorized as incident users. Early initiation and late HT initiation were defined using a 5-year cutoff as ≤5 and >5 years since menopause onset, respectively, or using a 10-year cutoff (≤10 and >10 years, respectively). The 5-year categorization and the 10-year categorization are the main categories used in previous literature. Therefore, we chose to use both. Considering that the category definitions are arbitrary, we also analysed the timing of initiation of HT as a continuous variable. For additional analyses of early HT initiation, women without available information about age at HT initiation and/or age at menopause onset were classified as early initiators based on their age at baseline: initiation of HT at ≤55 years for the 5-year cut-off and at ≤60 years for the 10-year cut-off.

HT was categorized as oestrogen-only or combined (oestrogen-progestin) HT. Women reporting the use of more than 1 HT medication were classified as users of combined HT if at least 1 of the medications was combined HT or 1 contained oestrogen alone and another contained progestin.

Oestradiol and CEEs were active ingredients of interest. All women who reported using both oestradiol and CEEs or neither of these medications (i.e., only other active ingredients) were excluded from the analysis that considered active ingredient.

Administration was considered to be oral if at least 1 of the medications was administered orally. In the absence of oral HT, administration was considered to be transdermal for at least 1 transdermally administered HT and vaginal in the absence of transdermal administration if 1 or more medications were vaginally administered. Vaginal HT could include creams, tablets, or rings.

The duration of use was classified, arbitrarily, as short (≤5 years) or long (>5 years).

The numbers of women in the various HT categories are shown in S1 Table, with information about data availability in each cohort.

### Definition of covariates

Based on age at baseline, 5 categories were defined: <55, 55–59, 60–64, 65–69, and ≥70 years of age. Age at menopause onset was divided into 3 categories: 41–46, 47–52 and 53–58 years of age. The level of education was classified as primary school, high school, or university. Body mass index (weight divided by height squared) was classified as <25, 25–30, or >30 kg/m^2^. Smoking status was classified as never, former, or current smoker. Parity was categorized as 0–1, 2–3, or ≥4 children and physical activity as low, moderate, or high intensity. Alcohol consumption was classified as low, moderate, or high, and oral contraceptive use as never or ever use.

Dyslipidaemia was ascertained from self-reports at baseline of use of lipid-lowering medication and/or a diagnosis of dyslipidaemia, as well as from the Swedish National Patient Register (ICD-9 code 272; ICD-10 code E78 at admission before the baseline investigation). Diabetes was ascertained from self-reported information at baseline; women who reported treatment with oral medication for diabetes or insulin were categorized as having diabetes. In addition, diabetes was identified using the Swedish National Patient Register (ICD-9 code 250; ICD-10 codes E10–E14). Hypertension was identified from self-reports of use of antihypertensive medication and/or a diagnosis of hypertension and from the Swedish National Patient Register (ICD-9 codes 401–404; ICD-10 codes I10–I13). Anatomical therapeutic chemical classification (ATC) codes considered likely to have been prescribed as medication for hypertension were C02, C03, C07, C08, and C09. Family history of CVD was defined as having at least 1 first-degree relative (parent or sibling) who experienced a myocardial infarct or stroke below the age of 60 years. The sources used to identify dyslipidaemia, diabetes, and hypertension were available for all cohorts, except WHILA (in which the questionnaire did not ask for information on dyslipidaemia).

### Statistical analysis

Information from the different cohorts was harmonized and mapped to a common set of variables. Stroke incidence and survival curves were compared between cohorts, before the pooling of data from individual participants in these cohorts, and were found to be fairly homogenous. Kaplan–Meier survival curves for the main exposure categories (early and late initiation and never use) were also plotted and found to be similar across cohorts; the early initiator category always had a better survival outcome (longer time free from a stroke event) than the never use category. The late initiator category sometimes had a better survival outcome than the never use category, but never a better survival outcome than the early initiator group. We considered that it was reasonable to combine the different cohorts, based on the population-based design of all 5 cohorts, the inclusion of women born during the same periods and therefore benefitting from the universal Swedish healthcare system, and the fact that the baseline investigations were all conducted at a time of a high HT prescription rate.

Participants contributed to time at risk from the date of the baseline investigation to the date of the first stroke (or haemorrhagic stroke where appropriate), the date of death from other causes, or the end of follow-up, whichever occurred first. Censored quantile regression, implemented using a Laplace regression [27] estimator, was applied to assess the potential associations between HT use and both incident stroke and haemorrhagic stroke, calculating the fifth percentile differences (PDs) and 95% confidence intervals (CIs). For the analysis of haemorrhagic stroke, the first PD was calculated instead. We calculated adjusted survival curves by estimating a grid of quantiles. The percentiles that could be reasonably estimated were selected based on the proportion of individuals with an event (i.e., those who were diagnosed with the disease) during the follow-up period; the proportions were 7.2% and 1.2% of the study population for stroke and haemorrhagic stroke, respectively. Estimating percentiles greater than the fifth for stroke and greater than the first for haemorrhagic stroke would require extrapolation beyond the range of observed data [28]. For analysis of the short-term risk of stroke associated with incident HT use, we also estimated the 0.5% PD, which roughly corresponds to the first 1.5 years of follow-up.

Laplace regression [27] is 1 of several techniques [29–31] for implementing censored quantile regression. It assumes that the outcome follows an asymmetric Laplace distribution but performs well under different data distributions. The technique provides robust estimates of the quantile regression coefficients [27,32].

For all analyses in the present study, the reference category was never users. The timing of HT initiation, as a continuous variable, was included in the regression models using restricted cubic splines with knots at the quartiles.

For several reasons, we decided to use Laplace regression rather than the more usual Cox regression. First, the hazard ratios (HRs) from Cox regression provide an overall estimate of relative risks based on assumptions of proportionality of hazards; in addition, less detailed information is available than from appropriately adjusted survival curves [33] such as estimates from Laplace regression [27,34]. Secondly, the use of survival curves enables better visualization of the results.

In order to calculate survival curves, a grid of percentiles (p1, p2, …) was estimated, and the estimated percentiles were plotted on the *x*-axis against the corresponding survival values given by (1 − p1), (1 − p2), …, on the *y*-axis. This approach is similar to creating Kaplan–Meier survival curves; however, an important difference is that estimates can be adjusted for covariates.

PDs are interpreted as follows. As an example, assuming that the fifth PD between groups A and B is 3 years, the survival curve of group A will take 3 years longer than that of group B to drop from 100% to 95%. In other words, the horizontal difference between the 2 curves, when survival is 95%, is 3 years (S2 Fig). In the results section, we referred generically to stroke-free period or haemorrhagic stroke-free period to indicate the percentiles or PDs. A PD point estimate of 0 corresponds to no difference in the disease-free period between the exposed and the reference group; thus, it is interpreted as a null result (corresponding to a HR of 1.0 had Cox regression been used).

We computed Kaplan–Meier curves describing the cumulative incidence of stroke and haemorrhagic stroke, respectively, cross stratifying by timing of HT initiation (using a 5-year cutoff) and each of the following variables: (1) age at baseline, (2) age at menopause onset, (3) level of education, (4) smoking status, (5) body mass index, (6) level of physical activity, (7) type of menopause, (8) parity, (9) use of oral contraceptives, (10) alcohol consumption, (11) presence of hypertension, (12) presence of dyslipidaemia, (13) presence of diabetes, (14) family history of CVD, and (15) cohort. Any marked differences between categories of these variables, as assessed visually, were further examined for potential confounding. The variables listed as 2–15 above were incorporated one at a time into crude models (adjusted only for age at baseline) for stroke and haemorrhagic stroke, respectively, separately modelling early and late HT initiation in relation to the outcome, to assess any substantial influence on the PD point estimates. The variables selected for inclusion in the final multivariable-adjusted model followed a prespecified criteria-based approach chosen on the basis of previous reports, visual inspection of the Kaplan–Meier curves, and any change ≥10% of the PD point estimates. Variables included in the final adjusted Laplace models were age at baseline, level of education, smoking status, body mass index, level of physical activity, and age at menopause onset.

A sensitivity analysis was conducted excluding 1 of each pair of twins in the SALT cohort to assess the robustness of the results. Of note, close relatives cannot be regarded as being sampled independently. In the case of monozygotic female twins as well as dizygotic twins who were both female, only 1 twin from each pair was included, randomly selected if both twins were either HT users or never users or otherwise the twin reporting HT use.

The Kaplan–Meier estimator was used to conduct further sensitivity analyses to examine whether stroke and haemorrhagic stroke associations varied between participants for whom detailed information on timing of HT initiation was or was not available. We also performed a complete case analysis, as a sensitivity analysis, to determine whether the exclusion of individuals due to missing values in the multivariable-adjusted final models is random.

All tests were 2-sided; a *P* value of 0.05 was considered statistically significant. Stata release 14 was used for all analyses. A prespecified analysis plan (S1 Text) was followed before first submission of the manuscript. After the peer-review process, a complete case analysis was added to address the issue of randomness of dropout due to missing data on covariates. Further, cohort as a covariate was added to the list of potential confounders evaluated (S2 Text).

This study is reported as per STROBE reporting guidelines (S1 STROBE Checklist).

## Results

Overall, 6,371 of the 88,914 (7%) postmenopausal women experienced an incident stroke over a median follow-up of 14.3 years. Altogether, 1,080 (17%) of these events were haemorrhagic stroke. The characteristics of study participants by HT use categories are shown in Table 1.

**Table 1:** Baseline characteristics of the postmenopausal women included in the present study of the Combined cohorts of menopausal women—Studies of register-based health outcomes in relation to hormonal drugs (COMPREHEND) material.

	Never user (*n* = 35,716)	Ever user (*n* = 53,198)	Early HT initiation* (*n* = 19,571)	Late HT initiation* (*n* = 7,189)	Total (*n* = 88,914)
Age at baseline investigation (years), median (IQR)	60.8 (56.5–68.9)	58.5 (53.3–63.2)	58.8 (54.8–60.5)	64.9 (60.0–70.2)	59.8 (54.5–65.8)
Age at menopause onset (years), median (IQR)	50.0 (48.0–52.2)	50.0 (48.0–52.1)	50.2 (48.0–53.0)	49.7 (46.0–52.0)	50.0 (48.0–52.2)
**Type of menopause (%)**					
Natural	86.8	82.3	80.2	80.9	84.1
Surgical^†^	13.2	17.7	19.8	19.1	15.9
**Smoking status (%)**					
Never	59.4	51.3	50.9	61.8	54.6
Former	20.4	28.2	28.6	24.6	24.9
Current	20.5	20.4	20.4	13.6	20.5
**Body mass index (kg/m**^**2**^**) (%)**					
<25	52.2	57.2	58.3	53.9	53.0
25–29.9	34.1	31.7	31.5	35.2	31.3
≥30	13.7	11.2	10.2	11.0	11.7
**Education (%)**					
Primary school	51.8	35.3	33.1	47.0	39.2
High school	33.2	40.8	40.5	36.0	35.5
University	15.0	23.9	26.4	17.0	19.2
**Physical activity (%)**					
Inactive	41.2	35.1	39.7	35.2	33.3
Moderate	46.5	48.6	46.1	51.0	42.5
Active	12.3	16.3	14.2	13.8	13.1
**Alcohol consumption (%)**					
Nondrinker	36.9	25.2	24.8	29.4	22.2
Moderate drinker	54.0	60.2	61.3	60.8	43.7
Heavy drinker	9.1	14.6	13.9	9.9	9.5
**Parity (number of children) (%)**					
0–1	26.9	26.4	23.6	27.2	26.3
2–3	59.6	63.6	64.9	60.9	61.4
≥4	13.5	10.0	11.5	12.0	11.3
Oral contraceptives, ever use (%)	37.6	52.8	57.0	32.5	41.6
Family history of cardiovascular disease (%)	17.2	16.8	19.4	18.1	11.1
Diabetes (%)	7.9	5.4	5.0	7.5	6.4
Hypertension (%)	23.6	24.2	23.0	28.9	23.9
Dyslipidaemia (%)	7.4	7.0	6.7	9.8	7.1

Data were missing for the following: smoking status, 0.9% of the participants; body mass index, 4.0%; education, 6.1%; physical activity, 11.1%; alcohol consumption, 24.6%; parity, 1.0%; oral contraceptives, 12.7%; and family history of cardiovascular disease, 34.6%.

*A 5-year cutoff was used to define early and late initiation groups.

†Menopause was considered surgical if menstruation ceased because of hysterectomy, oophorectomy, or gynaecological surgery (specified or unspecified).

HT, postmenopausal hormone therapy; IQR, interquartile range.

As shown in S2 Table, oestrogen-only HT and the vaginal route of administration, without taking into account timing of initiation, were associated with a longer stroke-free period than never use. Stroke and haemorrhagic stroke survival curves for route of administration are shown in S3 Fig. The remaining analyses presented in S2 Table show stroke-free or haemorrhagic stroke-free periods similar to those of never users or with wide CIs.

**Fig 1:**
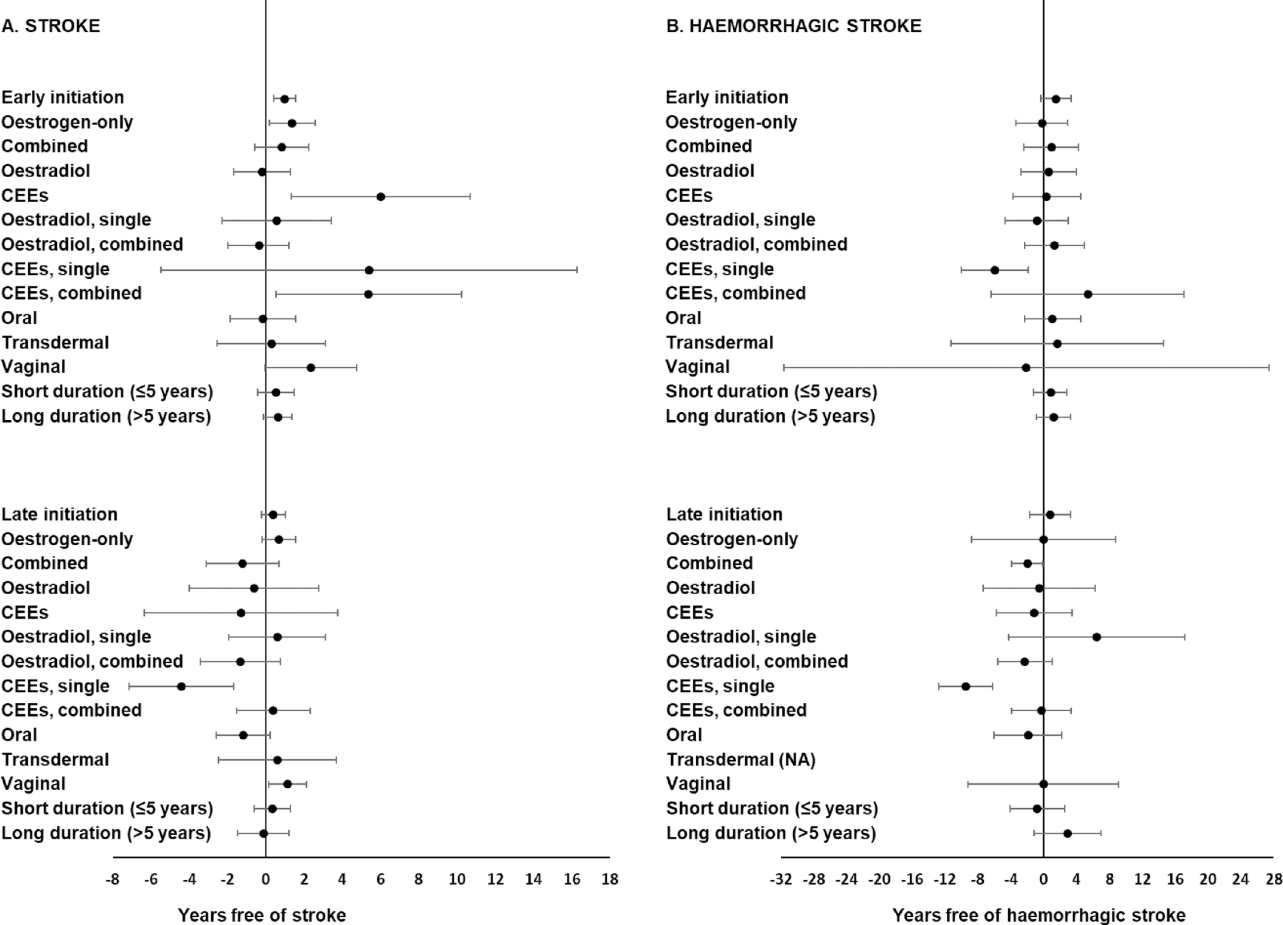
**Associations between multivariable-adjusted stroke-free (A) and haemorrhagic stroke-free (B) periods (percentile differences) and various categories of postmenopausal hormone therapy (HT) by timing of initiation using a 5-year cutoff.** The reference group is never use. The adjusted models included age at baseline, level of education (primary school, high school, or university), smoking status (never, former, or current), body mass index (<25, 25–29, or ≥30 kg/m^2^), level of physical activity (low, moderate, or high), and age at menopause onset (41–46, 47–52, or 53–58 years). The fifth and first percentile differences with 95% confidence intervals were calculated for stroke and haemorrhagic stroke, respectively. Note that the scale of the x-axis is different for stroke and haemorrhagic stroke. CEE, conjugated equine oestrogen; NA, not applicable, due to 0 haemorrhagic stroke cases among users of transdermal HT.

Results from analyses taking into account timing of HT initiation, employing the 5-year cutoff, are shown in Fig 1. The exact point estimate and confidence interval limits for each result are shown in S3 Table, which also includes results from analyses that employed the 10-year cutoff.

Analyses of both the timing of initiation and the type of HT revealed that early initiation of oestrogen-only HT was associated with a longer stroke-free period (Fig 1A). When initiated late with regard to menopause onset, combined HT use was associated with a shorter stroke-free (Fig 1A) and haemorrhagic stroke-free (Fig 1B, S3 Table) period than never use, although this finding was not statistically significant for stroke, regardless use of the 5-year or the 10-year cutoff. All other associations related to both timing of initiation and type of HT for stroke and haemorrhagic stroke were null or inconclusive (Fig 1 and S3 Table).

Simultaneous consideration of the timing of HT initiation and the active ingredient revealed that, if initiated early, CEE therapy was associated with a longer stroke-free period compared with never use (Fig 1A). Other analyses of HT timing and active ingredient showed stroke-free and haemorrhagic stroke-free periods similar to those of the never user group (Fig 1 and S3 Table).

Simultaneously considering the timing of HT initiation, the type, and the active ingredient showed that users of combined CEEs, if initiated early, had a longer stroke-free period than never users (Fig 1A and S3 Table); corresponding findings for haemorrhagic stroke were inconclusive (Fig 1B and S3 Table). Users of early, single CEE therapy had a shorter haemorrhagic stroke-free (Fig 1B) period than never users; corresponding findings for stroke were observed, but only when using the 10-year cutoff (S3 Table). The late use of single CEE therapy was associated with shorter stroke-free (Fig 1A) and haemorrhagic stroke-free (Fig 1B) periods; a shorter haemorrhagic stroke-free period was also observed for late use of combined oestradiol, but only when the 10-year cut-off was used (S3 Table). All other analyses of timing of initiation, HT type, and active ingredient produced PDs similar to those of never users or PD values with wide CIs (Fig 1 and S3 Table).

Analyses of both the route of administration and the timing of HT initiation showed that vaginal HT, regardless of its timing of initiation, was associated with a longer stroke-free period, although results were not statistically significant for early initiation (Fig 1A and S3 Table). Early initiation of vaginal HT was associated with a longer haemorrhagic stroke-free period when the 10-year cutoff was used (S3 Table). Other analyses of the route and timing of HT showed stroke-free and haemorrhagic stroke-free periods similar to those of never users or point estimates with wide CIs (Fig 1 and S3 Table). Results for haemorrhagic stroke concerning transdermal HT when initiated late could not be produced due to 0 cases among the users of transdermal HT.

Analyses of both the duration and the timing of initiation of HT provided null results (Fig 1 and S3 Table).

**Fig 2:**
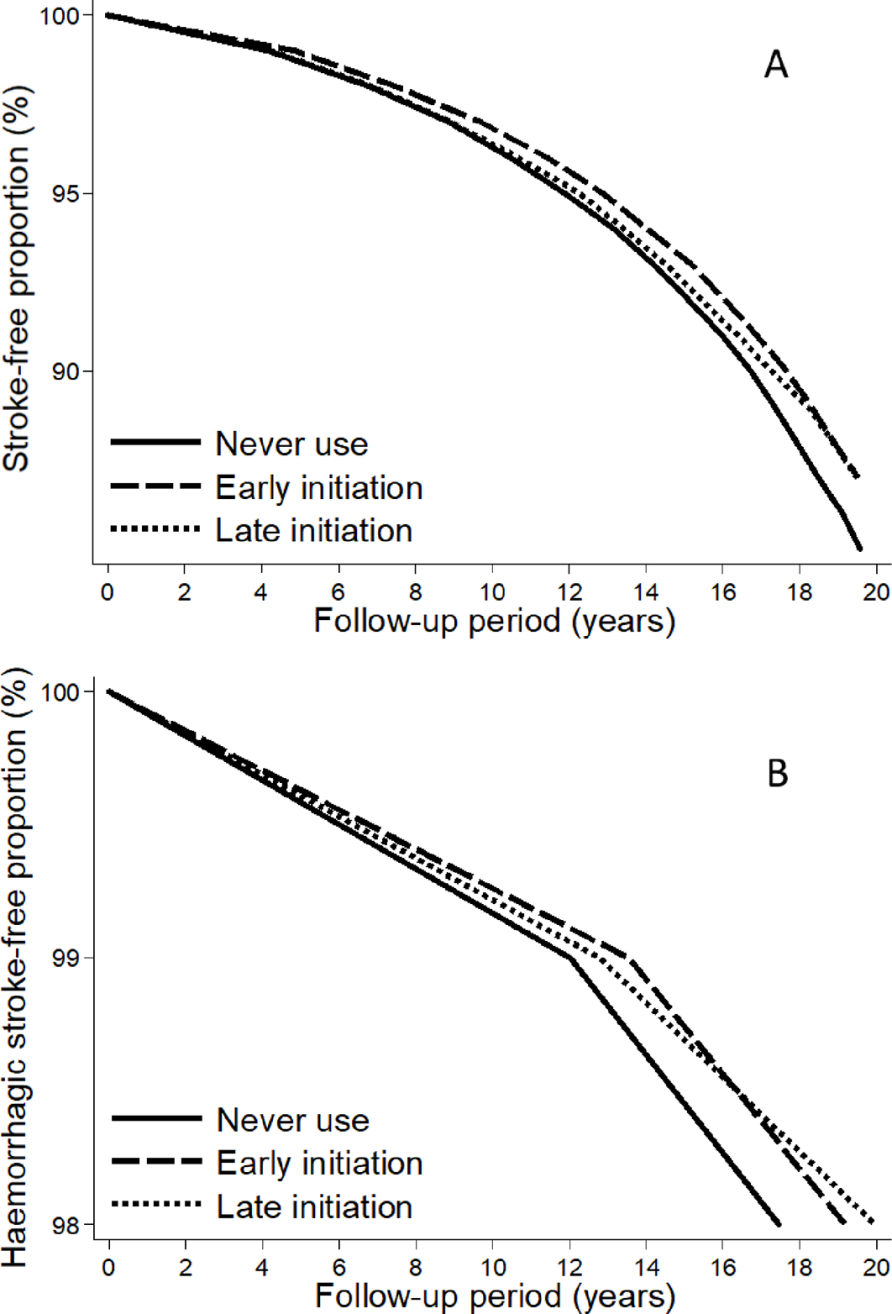
**Stroke (A) and haemorrhagic stroke (B) survival curves for early or late initiation, using the 5-year cutoff, of postmenopausal hormone therapy and never use.** The curves were computed using censored quantile regression adjusted for age at baseline, level of education, smoking status, body mass index, level of physical activity, and age at menopause onset.

**Fig 3:**
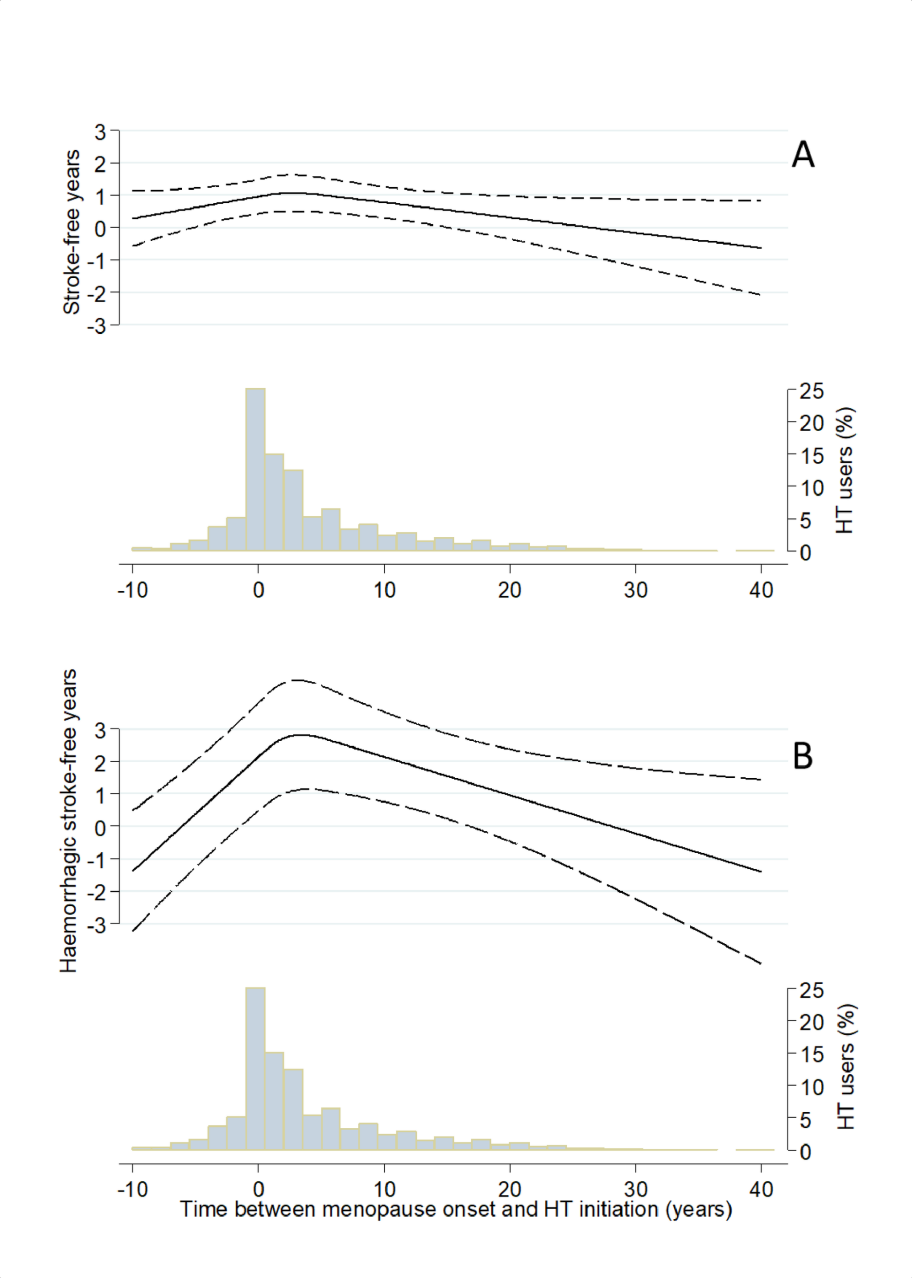
**Percentile difference (PD) between users and never users, modelled as a function of time of postmenopausal hormone therapy (HT) initiation using restricted cubic splines and adjusting for age at baseline, level of education, smoking status, body mass index, level of physical activity, and age at menopause onset: fifth PD of time-to-stroke (A) and first PD of time-to-haemorrhagic stroke (B**). Dashed lines represent pointwise 95% confidence intervals. The histograms illustrate the underlying distribution of timing of HT initiation. From (B), it can be seen that the PD between users and never users is at its maximum (about 3) when the time between menopause onset and HT initiation is approximately 5 years.

Early HT initiation, using the 5-year cutoff, was associated with a significantly longer stroke-free (Fig 1 and Fig 2A) period, but not with a significantly longer haemorrhagic stroke-free (Fig 1 and Fig 2B) period, than never use, after multivariable adjustments. When considering timing as a continuous variable, the stroke-free and haemorrhagic stroke-free periods were maximal if HT was initiated approximately 0–5 years from the onset of menopause (Fig 3A and 3B).

The results from the complete cases analysis revealed no pronounced discrepancies between crude results based on complete versus noncomplete cases (S4 Table).

In a sensitivity analysis, women without detailed information on timing of HT initiation were allocated to the early initiator groups based on their age at baseline for both the 5-year and 10-year cutoffs. The results from analyses based on these groups (S5 Table) generally support the interpretations of the main results shown in S3 Table. However, for single CEE therapy in relation to haemorrhagic stroke, results from the sensitivity analysis were inconclusive.

A comparison of results from analyses restricted to women with detailed information on timing and from analyses without this restriction showed no clear differences in either stroke risk or haemorrhagic stroke risk.

Analyses of incident use of HT (based on the 0.5% PD) did not show signs of elevated short-term stroke risk regardless of the timing of initiation, type, active ingredient, and route of administration of HT (S6 Table).

The sensitivity analysis including twins selected from the SALT cohort provided similar results to those obtained in the total study population (S7 Table).

## Discussion

In this population-based analysis of pooled individual participant data from more than 88,000 postmenopausal women, we observed no increased risk of stroke associated with HT when initiated <5 years since menopause onset. Overall, this finding persisted when the study outcome was restricted to haemorrhagic stroke. However, we observed increased risks of stroke and haemorrhagic stroke associated with late initiation of HT if it was administered with single CEE as the active ingredient. HT initiated late with regard to menopause onset and used in combination with a progestin was associated with an increased risk of haemorrhagic stroke only. Single CEE therapy was associated with an increased risk of haemorrhagic stroke regardless of timing of initiation.

Our finding that HT users had a similar stroke risk compared to never users is in agreement with the results from some observational studies [18], as well as from 2 RCTs: the Heart and Estrogen/Progestin Replacement Study (HERS) [3] and the Women's Estrogen for Stroke Trial (WEST) [4]. By contrast, the Nurses' Health Study (NHS) [20] and the Women's Health Initiative (WHI) [21,35] reported an increased risk of stroke, although neither timing of HT initiation nor regimens other than oral CEE therapy were considered. In all these studies—except WEST [4], in which a composite end point of ischaemic stroke and transient ischaemic attack was used—stroke was analysed as a composite end point of ischaemic stroke and haemorrhagic stroke.

Our findings on early HT initiation are not in agreement with those reported from a reanalysis of the NHS, where a positive association between HT (when initiated within 4 years from menopause onset) and stroke was observed: relative risk (RR) 1.29 (95% CI 1.06–1.58) and 1.22 (95% CI 0.95–1.55) for CEEs used as single and combined HT, respectively [14]. Reanalysis of the WHI [13] suggested that stroke risk did not depend on years since menopause at the time of HT initiation, but the results were not definite: HR 2.24 (95% CI 0.92–5.44) and 1.58 (95% CI 0.81–3.05) for oestrogen-only and combined HT, respectively, initiated within 10 years after the onset of menopause [13]. Our findings are in agreement with those from the Danish Osteoporosis Prevention Study (DOPS), in which women who had recently undergone menopause and were randomly assigned to receive combined treatment with oestradiol did not exhibit an increased risk of stroke (HR 0.77 [95% CI 0.35–1.70]) [9].

Our findings regarding late initiation of HT agree with results from the reanalysis of the NHS that showed an enhanced risk of stroke in association with CEEs as single therapy when initiated >10 years after menopause onset (RR 1.31 [95% CI 1.06–1.63]); the results regarding combined HT were not statistically significant (RR 1.18 [95% CI 0.87–1.60]) [14]. Reanalysis of the WHI indicated an enhanced risk of stroke in association with oestrogen-only (HR 1.47 [95% CI 0.92–2.35]) and combined CEEs (HR 1.12 [95% CI 0.76–1.64]) when initiated >10 years after the onset of menopause. Our finding that CEEs, but not oestradiol, were associated with an increased stroke risk in some analyses may reflect thrombotic effects exerted by CEEs [36,37]. In all of the aforementioned studies addressing the timing of HT initiation, women could not be precisely allocated into early and late HT initiation groups; allocation was based on age rather than detailed information on timing [10,13,14].

The majority of studies on HT and stroke risk have considered only oral administration [3,4,14,18,21]. However, among the different routes of administration, transdermal HT has been postulated as safer compared to oral HT with regard to stroke risk [38–41]. It was recently reported that oral oestrogen (odds ratio [OR] 1.58 [95% CI 1.01–2.49]), but not transdermal oestrogen (OR 0.83 [95% CI 0.56–1.24]), was associated with increased risk of ischaemic stroke [40]. In a register-based study that assessed vaginal HT, a reduced risk of death from stroke was reported [42]. To our knowledge, the present study is the first to investigate different routes of administration (oral, transdermal, and vaginal) while simultaneously considering the timing of HT initiation in relation to menopause onset.

Previous observational studies that analysed the association between oral HT and haemorrhagic stroke showed discrepant results [15–17,41]. To our knowledge, this is the first study to perform analyses considering the influence of the timing of HT initiation on the association between HT and haemorrhagic stroke. The reason for selecting haemorrhagic stroke as a separate end point was that stroke is a heterogeneous disease [43] with a complex pathogenesis [19]. Most previous studies analysed a stroke composite end point, probably due to an inability to analyse haemorrhagic stroke separately because of a limited number of clinical events. The results we observed for haemorrhagic stroke did not differ markedly from those for the composite end point, the latter mainly reflecting results for ischaemic stroke.

### Strengths and limitations

The present prospective study has important advantages, including the use of national high-quality registers, which provides the opportunity to follow cohort participants over time essentially without loss to follow-up, as well as valid identification of incident stroke and haemorrhagic stroke [44]. The detailed and extensive data available from the cohort participants enabled comprehensive analysis and control of potential confounding factors, including 15 different variables, most of them commonly used in previous literature. The detailed information on the timing of HT initiation provided by more than 26,000 HT users is unique. The present study considered more aspects of HT (time of initiation, type of therapy, route of administration, active ingredient, and duration) than did most previous studies in this area. Our findings should be generalizable across different geographical regions considering our population-based design, the large sample size, and a high internal validity. Finally, the large sample size allowed us to use novel statistical modelling methods suitable for addressing our research questions, with the results reported in a manner that may be more intuitive to interpret than that of standard methods [27,28].

Because of the observational design of this study, the possibility of uncontrolled confounding cannot be ruled out. It is likely that women who used HT during the time period of our baseline investigations were in general more health conscious and had a higher socioeconomic position [45]. However, various lifestyle factors as well as body mass index and level of education were considered. Although the presence of missing data on covariates could have influenced the results, the missing proportions for the variables included in our final adjusted model only varied between 0.9% and 11.1%. Furthermore, complete case analyses revealed no signs of selection bias. Although the possibility of immortal time bias, related to the observational design of the study [46], cannot be completely excluded, our results from analysis restricted to incident use do not support such bias. We have no information regarding whether or not women switched from exposed to unexposed status after the baseline investigations. This is a limitation that may have influenced our results, most probably towards a dilution.

Another limitation of our study is that we did not have detailed information about either the HT dose or the type of progestin used in the combined therapy, both of which have been discussed regarding a potential effect on stroke risk [14,40]. It is possible that our results concerning CEE when initiated late in relation to menopause onset would have been less pronounced had a lower dose been used. The recommended HT dose used in Sweden at the time when the women in our study were treated was 0.625 mg/day for CEE, 1–2 mg/day for oral oestradiol, and 50–100 μg/day for transdermal oestradiol [47]. The progestins predominantly used in Sweden during this period were nortestosterones (e.g., norethisterone acetate, levonorgestrel, and dionegest), although pregnanes (e.g., medroxyprogesterone acetate) were also used. Considering that derivatives of norpregnane, the progestin found to be associated with increased risk of ischaemic stroke in a previous study [40], were not frequently used in our study [48], the type of progestin is unlikely to explain our observed association between combined HT and risk of haemorrhagic stroke.

### Conclusion

HT was associated with a reduced or null risk of future stroke if initiated relatively soon after the onset of menopause, regardless of regimen (type, active ingredient, and route of administration) and duration. This finding was extended to haemorrhagic stroke, excepting use of CEEs as single therapy. However, when HT was initiated late with regard to menopause onset, an increased risk was observed for stroke and haemorrhagic stroke when CEEs were used as single therapy. Combined therapy initiated late was associated with increased risk of haemorrhagic stroke only.

Based on both our results and the available evidence from previous studies, we conclude that HT is not associated with increased stroke risk, if therapy is initiated soon after menopause onset. Our results suggest that the initiation of HT 0–5 years after menopause onset, as compared to never use, is associated with a decreased risk of stroke and haemorrhagic stroke.

## Supporting information

S1 STROBE Checklist Strengthening the reporting of observational studies in epidemiology (STROBE) checklist. (DOCX)

S1 Fig Postmenopausal women included in the study. (DOCX)

S2 Fig Survival curves computed using censored quantile regression. (DOCX)

S3 Fig Stroke and haemorrhagic stroke survival curves for route of administration. (DOCX)

S1 Table Number of women in different postmenopausal hormone therapy categories as assessed using the Combined cohorts of menopausal women—Studies of register-based health outcomes in relation to hormonal drugs (COMPREHEND) material. (DOCX)

S2 Table Stroke-free and haemorrhagic stroke-free periods in relation to the type, active ingredient, route of administration, and duration of postmenopausal hormone therapy, without stratification according to timing of initiation. Crude and multivariable-adjusted percentile differences are shown.
(DOCX)

S3 Table Stroke-free and haemorrhagic stroke-free periods in relation to the various categories of postmenopausal hormone therapy by timing of initiation. Crude and multivariable-adjusted percentile differences are shown.
(DOCX)

S4 Table Complete case analysis for stroke-free and haemorrhagic stroke-free periods in relation to the various categories of postmenopausal hormone therapy by timing of initiation. (DOCX)

S5 Table Sensitivity analysis in which women without detailed information on timing of postmenopausal hormone therapy initiation were allocated to the early initiator groups based on their age at baseline. (DOCX)

S6 Table Stroke-free periods in relation to the various categories of postmenopausal hormone therapy. Crude and multivariable-adjusted 0.5 percentile differences are shown. Ever use was restricted to incident use.
(DOCX)

S7 Table Sensitivity analysis including only 1 twin from each pair from the Screening Across the Lifespan Twin (SALT) cohort. (DOCX)

S1 Text Original study analysis plan. (DOCX)

S2 Text Modified study analysis plan. (DOCX)

## Abbreviations

CEE: conjugated equine oestrogen
CI: confidence interval
COMPREHEND: Combined cohorts of menopausal women—Studies of register-based health outcomes in relation to hormonal drugs
CVD: cardiovascular disease
DOPS: Danish Osteoporosis Prevention Study
HERS: Heart and Estrogen/Progestin Replacement Study
HR: hazard ratio
HT: postmenopausal hormone therapy
ICD: International Classification of Diseases
NHS: Nurses' Health Study
OR: odds ratio
PD: percentile difference
RCT: randomized controlled trial
SALT: Screening Across the Lifespan Twin
WEST: Women's Estrogen for Stroke Trial
WHI: Women's Health Initiative
