# Supplementary material for: Postmenopausal hormone therapy and risk of stroke: A pooled analysis of data from population-based cohort studies
Source: PLoS Med. 2017 Nov 17;14(11):e1002445. doi: 10.1371/journal.pmed.1002445 (PMC5693286; doi:10.1371/journal.pmed.1002445)
Supplement: S1 STROBE Checklist — (DOCX) [file pmed.1002445.s001.docx]

STROBE Statement—Checklist of items that should be included in reports of ***cohort studies***

|  | Item No | Recommendation | | Author comment | Manuscript excerpt(s) |
| --- | --- | --- | --- | --- | --- |
| **Title and abstract** | 1 | (*a*) Indicate the study’s design with a commonly used term in the title or the abstract | | The study design is stated in the Title and in the Abstract (Methods and Findings section). | *“Postmenopausal hormone therapy and risk of stroke: A pooled analysis of data from population-based cohort studies”*  *“…were combined in this observational study”* |
|  |  | (*b*) Provide in the abstract an informative and balanced summary of what was done and what was found | | The aim is stated at the end of the Background in the Abstract.  In the Methods and Findings section of the Abstract, we summarized how five pooled cohort studies were used to assess the study aim.  In the Conclusion section of the Abstract, we summarized what was found. | *“We aimed to assess the association between HT and risk of stroke, considering timing of initiation, route of administration, type, active ingredient, and duration of HT.”*  *“Data on HT use reported by the participants in five population-based Swedish cohort studies, with baseline investigations performed during the period 1987–2002, were combined in this observational study.”*  *“Laplace regression was employed to assess crude and multivariable-adjusted associations between HT and stroke risk by estimating percentile differences (PDs) with 95% confidence intervals (CIs). The 5th and 1st PDs were calculated for stroke and haemorrhagic stroke, respectively.”*  *“When initiated early in relation to menopause onset, HT was not associated with increased risk of incident stroke or haemorrhagic stroke, regardless of route of administration, type of HT, active ingredient, and duration. Our results suggest that the initiation of HT 0–5 years after menopause onset, as compared to never use, is associated with a decreased risk of stroke and haemorrhagic stroke. Late initiation of combined HT was associated with increased haemorrhagic stroke risk.”* |
| Introduction | | | |  |  |
| Background/rationale | 2 | | Explain the scientific background and rationale for the investigation being reported | The scientific background and rationale of the study are explained in the 1^st^ and 2^nd^ paragraph in the Background section. | *“However, subanalyses of this review concluded that there was no strong evidence on risk of stroke according to the timing of initiation of HT."*  *“…however the clinical relevance for hard endpoints such as stroke remains to be determined.”*  *“The importance of early initiation of HT in relation to stroke risk was addressed in a limited number of previous studies(9, 13, 14) and findings were inconsistent. Further, few studies have assessed the influence of HT use on the risk of haemorrhagic stroke, and results are inconclusive due to a limited number of cases and that timing of HT initiation was not considered. (15-18).”*  *“The importance of route of administration, type, active ingredient and duration of HT for stroke risk is unclear.(10) To our knowledge no previous studies have compared the different routes of administration (oral, transdermal or vaginal) and active ingredients (conjugated equine oestrogens [CEEs] or oestradiol) in relation to stroke risk.”*  *“The optimal duration of HT from the perspective of stroke risk also remains to be determined.(10)”* |
| Objectives | 3 | | State specific objectives, including any prespecified hypotheses | The specific objective is stated in the last paragraph of the Background section. | *“We aimed to assess the association between HT and the risk of developing stroke, considering haemorrhagic stroke separately, while taking into account timing of initiation of HT in relation to the onset of menopause, route of administration, type of HT, active ingredient and duration.”* |
| Methods | | | |  |  |
| Study design | 4 | | Present key elements of study design early in the paper | The key elements of the study design are presented in the last paragraph of the Background and in the 1^st^ paragraph of the Methods section. | *“Our analysis is based on combined data from a large number of postmenopausal women in five population-based Swedish cohorts.”*  *“This study is based on the COMPREHEND (Combined cohorts of menopausal women – studies of register-based health outcomes in relation to hormonal drugs)…”* |
| Setting | 5 | | Describe the setting, locations, and relevant dates, including periods of recruitment, exposure, follow-up, and data collection | In the Methods, we describe the setting, locations and relevant dates, including periods of recruitment, exposure, follow-up and data collection.  Every included cohort is explained in more detail under the subheading Description of cohorts in the Methods section.  The exposures in this study are described under the subheading Classification of HT in the Methods section. | *“…collaborative effort initiated in 2011.”*  *“Five cohorts included in COMPREHEND…Baseline investigations were performed between 1987 and 2002…”*  *“The end of follow-up varied between cohorts, from 31 December 2010 to 31 December 2013.”* |
| Participants | 6 | | (*a*) Give the eligibility criteria, and the sources and methods of selection of participants. Describe methods of follow-up | Source of participants, eligibility criteria and methods of follow-up are described in the Methods section. | *“…with available data on menopausal status, age at menopause onset, use of HT and age at HT initiation were invited to collaborate in the present study...”*  *“We excluded women with previous CVD…, as well as women who were not menopausal and those who were menopausal but reported an age at menopause outside the range of 40–59 years. After also excluding women for whom information on HT use was missing….”*  *“Utilizing the Swedish National Patient Register and the Swedish Cause of Death Register …during the follow-up period were identified.”* |
|  |  |  | (*b*) For matched studies, give matching criteria and number of exposed and unexposed | Not applicable |  |
| Variables | 7 | | Clearly define all outcomes, exposures, predictors, potential confounders, and effect modifiers. Give diagnostic criteria, if applicable | The exposures in this study are described under the subheading Classification of HT in the Methods section.  Predictors, potential confounders, and effect modifiers are described under the subheading Definition of Covariates in the Methods section. | *“…incident cases of stroke (ischaemic, haemorrhagic or unspecified; the International Classification of Diseases codes (ICD)-9 430–434/436–438, ICD-10 I60–I69) and of haemorrhagic stroke specifically (ICD-9 430–432, ICD-10 I60–I62)…”*  *“Main diagnoses and underlying causes of death were considered…”* |
| Data sources/ measurement | 8* | | For each variable of interest, give sources of data and details of methods of assessment (measurement). Describe comparability of assessment methods if there is more than one group | Sources of data for every included cohort are stated under the subheading Description of cohorts in the Methods section.  Sources of data for outcome are described under the subheading Follow-up and definition of endpoints in the Methods section. | *“Five cohorts included in COMPREHEND and with available data (from questionnaire or interviews) on menopausal status, age at menopause onset, use of HT and age at HT initiation were invited to collaborate in the present study and all agreed. The cohorts are described below. Baseline investigations were performed between 1987 and 2002, a period during which HT was frequently prescribed. …”*  *“Utilizing the Swedish National Patient Register and the Swedish Cause of Death Register, incident cases of stroke…”* |
|  |  | |  |  |  |
| Bias | 9 | | Describe any efforts to address potential sources of bias | The strategy for confounding control is explained in the Methods. The analysis of incident HT use, to address immortal time bias, is described in the Methods section. | *“The variables selected for inclusion in the final multivariable-adjusted model followed a pre-specified criteria-based approach chosen on the basis of previous reports, visual inspection of the Kaplan–Meier curves and any change ≥10% of the PD point estimates.”*  *“For analysis of the short-term risk of stroke associated with incident HT use …”* |
| Study size | 10 | | Explain how the study size was arrived at | The sample size was governed by the availability of data in the COMPREHEND, after applying the eligibility criteria explained above in item No 6(a).  A flow-chart (Figure 1) in the Methods section describes the inclusion of women into the present study. | *“…88,914 were included in the study (S1 Fig) (i.e.30,832, 20,108, 18,818, 13,700, 5,456 from the SMC, NSHDS, SALT, MDCS and WHILA cohort,, respectively.”* |
| Quantitative variables | 11 | | Explain how quantitative variables were handled in the analyses. If applicable, describe which groupings were chosen and why | The variables and grouping are explained under the subheadings Classification of HT and Definition of covariates in the Methods section. | *“Women who reported current or previous use of HT were categorized as ever users. Ever users who reported initiating HT within the previous 12 months were categorized as incident users. Early and late HT initiation were defined using a 5-year cut-off as ≤5 and >5 years since menopause onset, respectively, or using a 10-year cut-off (≤10 and >10 years respectively). The 5-year and 10-year categorization are the main categories used in previous literature. Therefore, we chose to use both. Considering that the category definitions are arbitrary, we also analysed the timing of initiation of HT as a continuous variable. …”* |
| Statistical methods | 12 | | (*a*) Describe all statistical methods, including those used to control for confounding | All statistical methods are described under the subheading Statistical analyses in the Methods section, including those used to control for confounding. | *“We computed Kaplan–Meier curves describing the cumulative incidence of stroke and haemorrhagic stroke, respectively, cross-stratifying by timing of HT initiation (using a 5-year cut-off) and each of the following variables… Substantial differences between categories of these variables, assessed by visual inspection, were subject to further examination for potential confounding.”*  *“Censored quantile regression, implemented using Laplace regression(27) estimator, was applied to assess the potential associations between HT use and both incident stroke and haemorrhagic stroke, calculating the 5th percentile differences (PDs) and 95% confidence intervals (CIs). For the analysis of haemorrhagic stroke, the 1st PD was calculated instead.”*  *“The variables listed as 2–15 above were incorporated one at a time into crude models (adjusted only for age at baseline) for stroke and haemorrhagic stroke, respectively, separately modelling eary and late HT initiation in relation to the outcome, to assess any substantial influence on the PD point estimates. The variables selected for inclusion in the final multivariable-adjusted model followed a pre-specified criteria-based approach chosen on the basis of previous reports, visual inspection of the Kaplan–Meier curves and any change ≥10% of the PD point estimates.”* |
|  |  |  | (*b*) Describe any methods used to examine subgroups and interactions | No stratifications were made and interactions were not assessed. |  |
|  |  |  | (*c*) Explain how missing data were addressed | To address missing data concerning timing of HT initiation, a sensitivity analysis was performed, described under the subheading Statistical analyses in the Methods section. | *“The Kaplan–Meier estimator was used to conduct further sensitivity analyses to examine whether stroke and haemorrhagic stroke associations varied between participants for whom detailed information on timing of HT initiation was or was not available. We also performed a complete case analysis, as a sensitivity analysis, to determine whether the exclusion of individuals due to missing values in the multivariable-adjusted final models is random.”* |
|  |  |  | (*d*) If applicable, explain how loss to follow-up was addressed | The loss to follow-up is virtually inexistent as explained under the subheading Strengths and limitations in the Discussion section. | *“use of national high-quality registers, which provides the opportunity to follow cohort participants over time essentially without loss to follow-up”* |
|  |  |  | (*e*) Describe any sensitivity analyses | Sensitivity analyses are described under the subheading Statistical analyses in the Methods section and at the end of the Results section. | *“A sensitivity analysis was conducted excluding one of each pair of twins in the SALT cohort to assess the robustness of the results. Of note, close relatives cannot be regarded as being sampled independently. In the case of monozygotic female twins as well as dizygotic twins who were both female, only one twin from each pair was included, randomly selected if both twins were either HT users or never users or otherwise the twin reporting HT use.”*  *“The Kaplan–Meier estimator was used to conduct further sensitivity analyses to examine whether stroke and haemorrhagic stroke associations varied between participants for whom detailed information on timing of HT initiation was or was not available. We also performed a complete case analysis, as a sensitivity analysis, to determine whether the exclusion of individuals due to missing values in the multivariable-adjusted final models is random.”*  *“In a sensitivity analysis, women without detailed information on timing of HT initiation were allocated to the early initiator groups based on their age at baseline for both the 5-year and 10-year cut-offs.”* |
| Results | | | |  |  |
| Participants | 13* | | (a) Report numbers of individuals at each stage of study—eg numbers potentially eligible, examined for eligibility, confirmed eligible, included in the study, completing follow-up, and analysed | S1 Fig is a flowchart giving the required information. | *“S1 Fig caption*  *Postmenopausal women included in the study.”* |
|  |  |  | (b) Give reasons for non-participation at each stage |  |  |
|  |  |  | (c) Consider use of a flow diagram |  |  |
| Descriptive data | 14* | | (a) Give characteristics of study participants (eg demographic, clinical, social) and information on exposures and potential confounders | In the Results section, characteristics of study participants by exposure category are given in Table 1. | *“Table 1. Baseline characteristics of the postmenopausal women included in the present study of the COMPREHEND material.”* |
|  |  |  | (b) Indicate number of participants with missing data for each variable of interest | The proportions with missing data on covariates are given in Table 1. | *“Data missing for: smoking status 0.9%, body mass index 4.0%, education 6.1%, physical activity 11.1, alcohol consumption 24.6%, parity 1.0%, oral contraceptives 12.7% and family history of cardiovascular disease 34.6% of the participants.”* |
|  |  |  | (c) Summarise follow-up time (eg, average and total amount) | The mean follow-up time is stated in the first sentence in the Results section. | *“…over a median follow-up of 14.3 years...”* |
| Outcome data | 15* | | Report numbers of outcome events or summary measures over time | The number of outcome events is given in the text in the Results section and in tables: Table 3, Table 4 and Table 5. | *“Overall, 6,371 of the 88,914 (7%) postmenopausal women experienced and incident stroke over a median follow-up of 14.3 years. Overall, 1,080 (17%) of these events were haemorrhagic stroke.”* |
| Main results | 16 | | (*a*) Give unadjusted estimates and, if applicable, confounder-adjusted estimates and their precision (eg, 95% confidence interval). Make clear which confounders were adjusted for and why they were included | In the Results section tables, we report both crude and adjusted estimates along with 95% confidence intervals, as indicated in the footnotes.  The reasons for including the potential confounders in the model are explained above under Item No 12(a). | *“Crude model was adjusted for age at baseline only (<55, 55–59, 60–64, 65–69 or ≥70 years). The adjusted model included age at baseline, level of education (primary school, high school or university), smoking status (never, former or current), body mass index (<25, 25–29 or ≥30 kg/m2), level of physical activity (low, moderate or high) and age at menopause onset (41–46, 47–52 or 53–58 years).”* |
|  |  |  | (*b*) Report category boundaries when continuous variables were categorized |  |  |
|  |  |  |  |  |  |
|  |  |  | (*c*) If relevant, consider translating estimates of relative risk into absolute risk for a meaningful time period | Not considered relevant. |  |
| Other analyses | 17 | | Report other analyses done—eg analyses of subgroups and interactions, and sensitivity analyses | Sensitivity analysis were done as described above under Item No 12(e).  We performed analyses where HT use was restricted to incident HT use, as explained in the Methods, Results and Discussion sections. | *“Analyses of incident use of HT (based on the 0.5% PD) did not show signs of elevated short-term stroke risk regardless of timing of initiation, type, active ingredient and route of administration of HT (S6 Table).”*  *“Although the possibility of immortal time bias, related to the observational design of the study, [*[*45*](#_ENREF_45)*] cannot be completely excluded, our results from analysis restricted to incident use do not support such bias.”* |
| Discussion | | | |  |  |
| Key results | 18 | | Summarise key results with reference to study objectives | Key results are summarized in the first paragraph in the Discussion section, directly connecting them to our study objectives. | *“…we observed neither an increased nor a decreased risk of stroke associated with HT when initiated early in relation to menopause onset and used as oestrogen-only HT. This finding persisted when the study outcome was restricted to haemorrhagic stroke. However, we observed increased risks of stroke and haemorrhagic stroke associated with late initiation of HT if administered as single CEE as the active ingredient. HT initiated late with regard to menopause onset and used in combination with a progestin was associated with an increased risk of haemorrhagic stroke only. Single CEE therapy was associated with increased risk of haemorrhagic stroke regardless of timing of initiation”* |
| Limitations | 19 | | Discuss limitations of the study, taking into account sources of potential bias or imprecision. Discuss both direction and magnitude of any potential bias | Under the Strengths and limitations subheading in the Discussion section we discuss the limitations of the study. | *“The possibility of uncontrolled confounding cannot be ruled out, because of the observational design of this study. It has been suggested that women who used HT were in general more health-conscious and had a higher socioeconomic position.[45] However, various lifestyle factors as well as body mass index and level of education were considered. Although the presence of missing data on covariates could have influenced the results, the missing proportions for the variables included in our final adjusted model only varied between 0.9% and 11.1%. Furthermore, complete case analyses revealed no signs of selection bias. Although the possibility of immortal time bias, related to the observational design of the study, [46] cannot be completely excluded our results from analysis restricted to incident use do not support such bias. We have no information regarding whether or not women switched from exposed to unexposed status after the baseline investigations. This is a limitation that may have influenced our results, most probably towards a dilution.”*  *“Another limitation of our study is that we did not have detailed information about either HT dose or type of progestin used in the combined therapy, both which of have been discussed regarding a potential effect on stroke risk. [*[*14*](#_ENREF_14)*,* [*40*](#_ENREF_40)*] It is possible that our results concerning CEE when initiated late in relation to menopause onset would have been less pronounced had a lower dose been used.”* |
| Interpretation | 20 | | Give a cautious overall interpretation of results considering objectives, limitations, multiplicity of analyses, results from similar studies, and other relevant evidence | In the Conclusion section, we state the overall interpretation of results considering objectives, limitations and results from similar studies. | *“HT was associated with a reduced or null risk of future stroke or haemorrhagic stroke if initiated relatively soon after the onset of menopause, regardless of regimen (type, active ingredient and route of administration) and duration. However, when HT was initiated late with regard to menopause onset, an increased risk was observed for stroke and haemorrhagic stroke when CEEs were used as single therapy. Combined therapy initiated late was associated with increased risk of haemorrhagic stroke only”.*  *“Based on both our results and the available evidence from previous studies, we conclude that HT is not associated with increased stroke risk, if therapy is initiated soon after menopause onset. Our results suggest that the initiation of HT 0–5 years after menopause onset, as compared to never use, is associated with a decreased risk of stroke and haemorrhagic stroke. Although not statistically significant, our results lend some support to previous suggestions that non-oral routes are safer from the perspective of stroke risk.”* |
| Generalisability | 21 | | Discuss the generalisability (external validity) of the study results | In the Discussion section, under the Strengths and limitations subheading, we discuss the generalisability. | *“Our findings should be generalizable across different geographical regions considering our population-based design, the large sample size and a high internal validity.”* |
| Other information | | | |  |  |
| Funding | 22 | | Give the source of funding and the role of the funders for the present study and, if applicable, for the original study on which the present article is based | This information is provided. | *“Funding sources: Financial support to extract register data, pool data from the collaborative cohorts, perform the analyses and draft the manuscript was received from Karolinska Institutet (Junior Faculty Support at the Institute of Environmental Medicine to K.L.). The collaborative cohorts are supported financially by grants from the Swedish Research Council, the Swedish Heart-Lung Foundation and the Local County Councils of Norrbotten and Västerbotten.”*  *“Role of the funding sources: The funders had no role in the study design, data collection, data analysis, data interpretation or writing of the report.* |

*Give information separately for exposed and unexposed groups.

**Note:** An Explanation and Elaboration article discusses each checklist item and gives methodological background and published examples of transparent reporting. The STROBE checklist is best used in conjunction with this article (freely available on the Web sites of PLoS Medicine at http://www.plosmedicine.org/, Annals of Internal Medicine at http://www.annals.org/, and Epidemiology at http://www.epidem.com/). Information on the STROBE Initiative is available at http://www.strobe-statement.org.
