# Supplementary figures and images for: Postmenopausal hormone therapy and risk of stroke: A pooled analysis of data from population-based cohort studies
Source: PLoS Med. 2017 Nov 17;14(11):e1002445. doi: 10.1371/journal.pmed.1002445 (PMC5693286; doi:10.1371/journal.pmed.1002445)

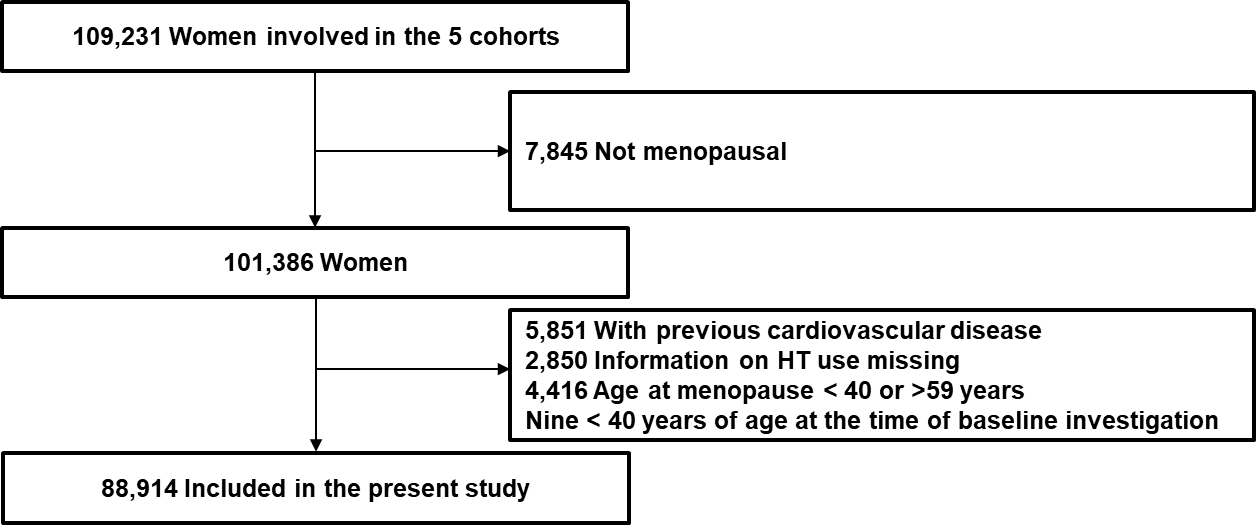


**S1 Fig.** Postmenopausal women included in the study.

Supplement: S1 Fig — (DOCX) [file pmed.1002445.s002.docx]
