# Supplementary material for: Postmenopausal hormone therapy and risk of stroke: A pooled analysis of data from population-based cohort studies
Source: PLoS Med. 2017 Nov 17;14(11):e1002445. doi: 10.1371/journal.pmed.1002445 (PMC5693286; doi:10.1371/journal.pmed.1002445)
Supplement: S2 Fig — (DOCX) [file pmed.1002445.s003.docx]

**S2 Fig.** Survival curves computed using censored quantile regression (Laplace regression) on a grid of quantiles, showing the event-free proportion in groups A and B. For example, survival at time t = 15 years was 95% in group A, which also means that t = 15 is the 5^th^ percentile of survival in this group. Analogously, in group B, the 5^th^ percentile of survival is t = 18 years, which yields a 5^th^ PD of 3. This value corresponds to the horizontal difference between the two survival curves
