# Supplementary material for: Postmenopausal hormone therapy and risk of stroke: A pooled analysis of data from population-based cohort studies
Source: PLoS Med. 2017 Nov 17;14(11):e1002445. doi: 10.1371/journal.pmed.1002445 (PMC5693286; doi:10.1371/journal.pmed.1002445)
Supplement: S3 Fig — (DOCX) [file pmed.1002445.s004.docx]

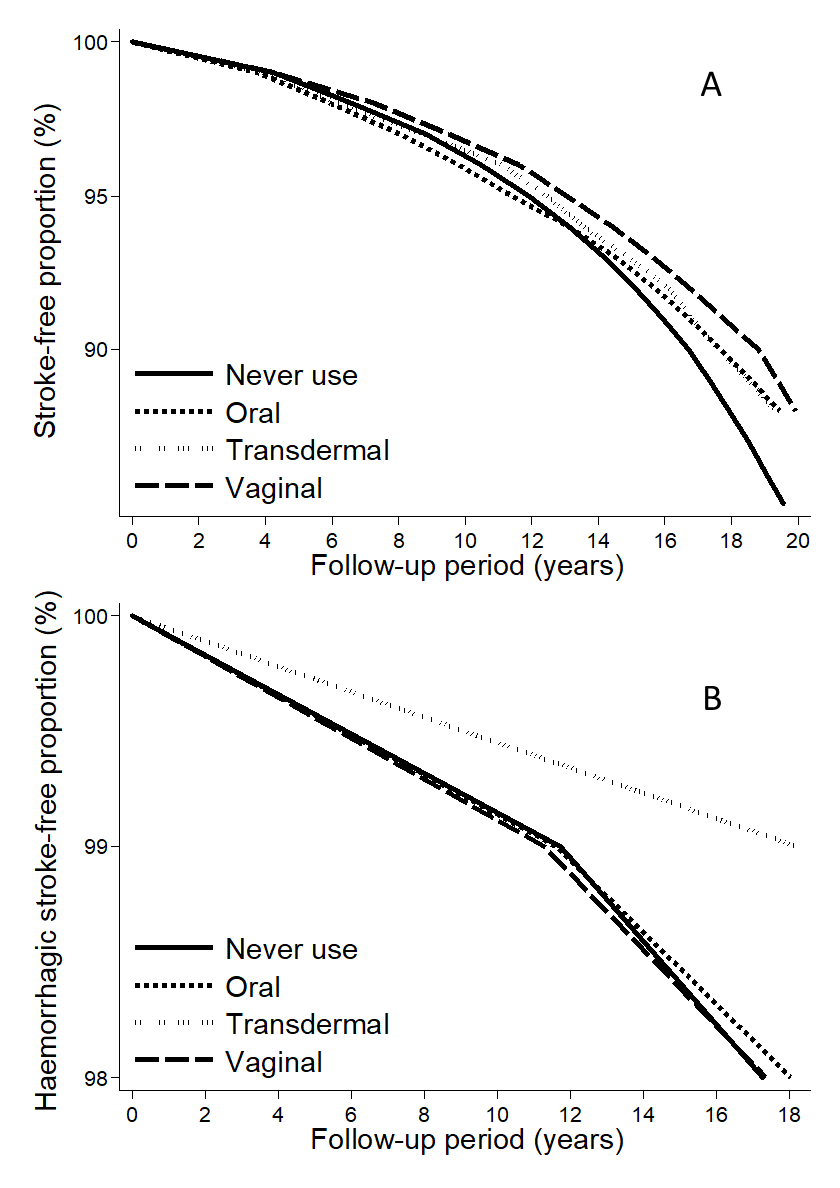


**S3 Fig.** Stroke (A) and haemorrhagic stroke (B) survival curves for route of administration (oral, transdermal, and vaginal) of postmenopausal hormone therapy and never use. The curves were computed using censored quantile regression adjusted for age at baseline, level of education, smoking status, body mass index, level of physical activity, and age at menopause onset.
