# Supplementary material for: Postmenopausal hormone therapy and risk of stroke: A pooled analysis of data from population-based cohort studies
Source: PLoS Med. 2017 Nov 17;14(11):e1002445. doi: 10.1371/journal.pmed.1002445 (PMC5693286; doi:10.1371/journal.pmed.1002445)
Supplement: S1 Table — (DOCX) [file pmed.1002445.s005.docx]

| **S1 Table. Number of women in different postmenopausal hormone therapy categories as assessed using the COMPREHEND material.** | |
| --- | --- |
|  | N |
| HT ever use | 53,198 |
| Never use | 35,716 |
| Detailed information on timing of HT initiation | 26,760 |
| Incident use of HT | 3,862 |
| **Type of HT** |  |
| Oestrogen-only | 8,184 |
| Combined | 9,705 |
| **Active ingredient** |  |
| Oestradiol | 10,848 |
| CEEs | 1,691 |
| **Type and active ingredient of HT** |  |
| Single oestradiol | 3,502 |
| Combined oestradiol | 7,346 |
| Single CEEs | 605 |
| Combined CEEs | 1,086 |
| **Route of administration** |  |
| Oral | 8,760 |
| Transdermal | 1,985 |
| Vaginal | 1,969 |
| **Duration of HT** |  |
| ≤5 years | 18,419 |
| >5 years | 10,283 |
| In the Malmö Diet and Cancer Study, only 20% of the women were asked to provide information allowing detailed assessment of timing. Information on type, active ingredient and route of administration was not available for the Swedish Mammography Cohort. No information on the route of administration was available for the Northern Sweden Health and Disease Study; in addition, vaginal route could not be examined in the Women’s Health in the Lund Area cohort.  COMPREHEND: Combined cohorts of menopausal women – studies of register-based health outcomes in relation to hormonal drugs, HT: postmenopausal hormone therapy, CEEs: conjugated equine oestrogens. | |
