# Supplementary material for: Postmenopausal hormone therapy and risk of stroke: A pooled analysis of data from population-based cohort studies
Source: PLoS Med. 2017 Nov 17;14(11):e1002445. doi: 10.1371/journal.pmed.1002445 (PMC5693286; doi:10.1371/journal.pmed.1002445)
Supplement: S2 Table — Crude and multivariable-adjusted percentile differences are shown. (DOCX) [file pmed.1002445.s006.docx]

| **S2 Table. Stroke-free and haemorrhagic stroke-free periods in relation to the type, active ingredient, route of administration and duration of postmenopausal hormone therapy, without stratification women according to timing of initiation. Crude and multivariable-adjusted percentile differences are shown.** | | | | |
| --- | --- | --- | --- | --- |
|  | **N** | **Crude^a^**  PD (95% CI) | **N** | **Adjusted^a^**  PD (95% CI) |
| **Type of HT (oestrogen-only or combined)** | 53,605 |  | 38,627 |  |
| **Never use** | 35,716 | 0 (Reference) | 26,905 | 0 (Reference) |
| **Oestrogen-only HT** | 8,184 |  | 5,232 |  |
| Stroke |  | 1.36 (0.70, 2.03)* |  | 0.95 (0.29, 1.61)* |
| Haemorrhagic stroke |  | 0.86 (-1.96, 3.68) |  | 0.08 (-2.11, 2.27) |
| **Combined HT** | 9,705 |  | 6,490 |  |
| Stroke |  | 0.98 (0.10, 1.85)* |  | 0.28 (-0.79, 1.34) |
| Haemorrhagic stroke |  | 0.72 (-0.87, 2.30) |  | -0.49 (-2.84, 1.86) |
| **Active ingredient** | 48,255 |  | 34,760 |  |
| **Never use** | 35,716 | 0 (Reference) | 26,905 | 0 (Reference) |
| **Oestradiol** | 10,848 |  | 6,819 |  |
| Stroke |  | 0.78 (-0.15, 1.71) |  | 0.03 (-1.29, 1,35) |
| Haemorrhagic stroke |  | 0.70 (-0.98, 2.38) |  | 0.45 (-2.04, 2.95) |
| **CEEs** | 1,691 |  | 1,036 |  |
| Stroke |  | 2.85 (0.70, 5.00)* |  | 1.62 (-2.10, 5.34) |
| Haemorrhagic stroke |  | 2.62 (-1.63, 6.86) |  | -0.50 (-6.19, 5.19) |
| **Route of administration** | 48,430 |  | 35,253 |  |
| **Never use** | 35,716 | 0 (Reference) | 26,905 | 0 (Reference) |
| **Oral** | 8,760 |  | 5,604 |  |
| Stroke |  | 0.41 (-0.46, 1.28) |  | -0.52 (-1.73, 0.70) |
| Haemorrhagic stroke |  | 0.18 (-2.54, 2.90) |  | -0.10 (-1.87, 1.67) |
| **Transdermal** | 1,985 |  | 991 |  |
| Stroke |  | 1.59 (-0.31, 3.49) |  | 0.55 (-1.24, 2.33) |
| Haemorrhagic stroke |  | 4.23 (-2.11, 10.57) |  | 6.51 (-1.41, 14.42) |
| **Vaginal** | 1,969 |  | 1,753 |  |
| Stroke |  | 1.68 (0.56, 2.81)* |  | 1.13 (0.06, 2.21)* |
| Haemorrhagic stroke |  | 0.87 (-2.57, 4.31) |  | -0.44 (-2.75, 1.88) |
| **Duration of HT** | 64,418 |  | 46,301 |  |
| **Never use** | 35,716 | 0 (Reference) | 26,905 | 0 (Reference) |
| **≤5 years** | 18,419 |  | 12,002 |  |
| Stroke |  | 1.03 (0.48, 1.57)* |  | 0.67 (0.06, 1.29)* |
| Haemorrhagic stroke |  | 1.32 (-0.42, 3.06) |  | 0.42 (-1.24, 2.07) |
| **>5 years** | 10,283 |  | 7,394 |  |
| Stroke |  | 0.51 (-0.01, 1.03) |  | 0.44 (-0.15, 1.03) |
| Haemorrhagic stroke |  | 1.06 (-0.82, 2.95) |  | 1.58 (-1.21, 4.36) |
| **^a^**Crude model was adjusted for age at baseline only (<55, 55–59, 60–64, 65–69 or ≥70 years). The adjusted model included age at baseline, level of education (primary school, high school or university), smoking status (never, former or current), body mass index (<25, 25–29 or ≥30 kg/m^2^), level of physical activity (low, moderate or high) and age at menopause onset (41–46, 47–52 or 53–58 years). The 5^th^ and 1^st^ percentile differences along with 95% confidence intervals, were calculated for stroke and haemorrhagic stroke, respectively.  P-values smaller than 0.05 are indicated with an asterisk (*)  PD: percentile differences, CI: confidence interval, HT: postmenopausal hormone therapy, CEE: conjugated equine oestrogen. | | | | |
