# Supplementary material for: Postmenopausal hormone therapy and risk of stroke: A pooled analysis of data from population-based cohort studies
Source: PLoS Med. 2017 Nov 17;14(11):e1002445. doi: 10.1371/journal.pmed.1002445 (PMC5693286; doi:10.1371/journal.pmed.1002445)
Supplement: S3 Table — Crude and multivariable-adjusted percentile differences are shown. (DOCX) [file pmed.1002445.s007.docx]

| **S3 Table. Stroke-free and haemorrhagic stroke-free periods in relation to the various categories of postmenopausal hormone therapy by timing of initiation. Crude and multivariable-adjusted percentile differences are shown.** | | | | | | | | |
| --- | --- | --- | --- | --- | --- | --- | --- | --- |
|  | **Early and late HT initiation: 5-year cut-off** | | | | **Early and late HT initiation: 10-year cut-off** | | | |
|  | **N** | **Crude^a^**  PD (95% CI) | **N** | **Adjusted^a^**  PD (95% CI) | **N** | **Crude^a^**  PD (95% CI) | **N** | **Adjusted^a^**  PD (95% CI) |
| **Timing of HT initiation** | 62,476 |  | 49,956 |  | 62,476 |  | 49,956 |  |
| **Never use** | 35,716 | 0 (Reference) | 26,905 | 0 (Reference) | 35,716 | 0 (Reference) | 26,905 | 0 (Reference) |
| **Early initiation** | 19,571 |  | 17,101 |  | 22,983 |  | 20,054 |  |
| Stroke |  | 1.02 (0.51, 1.53)* |  | 1.00 (0.42, 1.57)* |  | 0.93 (0.42, 1.43)* |  | 0.71 (0.18, 1.24)* |
| Haemorrhagic stroke |  | 2.24 (0.26, 4.22)* |  | 1.52 (-0.32, 3.37) |  | 2.18 (0.53, 3.84)* |  | 1.51 (-0.38, 3.40) |
| **Late initiation** | 7,189 |  | 5,950 |  | 3,777 |  | 2,997 |  |
| Stroke |  | 0.82 (0.12, 1.52)* |  | 0.38 (-0.24, 1.01) |  | 1.08 (0.39, 1.77) * |  | 0.59 (-0.20, 1.39) |
| Haemorrhagic stroke |  | 1.79 (0.15, 3.42)* |  | 0.77 (-1.72, 3.26) |  | 1.78 (-0.67, 4.23) |  | 0.64 (-1.82, 3.11) |
| **Type and timing of HT** | 48,093 |  | 37,682 |  | 48,093 |  | 37,682 |  |
| **Never use** | 35,716 | 0 (Reference) | 26,905 | 0 (Reference) | 35,716 | 0 (Reference) | 26,905 | 0 (Reference) |
| **Oestrogen-only, early** | 3,243 |  | 2,774 |  | 4,257 |  | 3,590 |  |
| Stroke |  | 1.57 (0.07, 3.06)* |  | 1.38 (0.18, 2.58)* |  | 1.43 (0.34, 2.52)* |  | 1.12 (-0.07, 2.31) |
| Haemorrhagic stroke |  | 0.04 (-2.27, 2.36) |  | -0.20 (-3.34, 2.94) |  | -0.43 (-3.11, 2.25) |  | -0.71 (-5.26, 3.85) |
| **Oestrogen-only, late** | 2,459 |  | 1,844 |  | 1,445 |  | 1,028 |  |
| Stroke |  | 1.42 (0.43, 2.41)* |  | 0.67 (-0.20, 1.55) |  | 1.67 -0.68, 2.65) |  | 0.99 (0.26, 1.72)* |
| Haemorrhagic stroke |  | 1.32 (-14.27, 16.91) |  | -0.03 (-8.80, 8.73) |  | 2.64 (0.88, 4.40)* |  | 2.23 (-3.14, 7.59) |
| **Combined, early** | 5,469 |  | 5,072 |  | 6,264 |  | 5,813 |  |
| Stroke |  | 1.28 (0.22, 2.34) * |  | 0.83 (-0.57, 2.24) |  | 1.16 (0.09, 2.22)* |  | 0.55 (-0.85, 1.95) |
| Haemorrhagic stroke |  | 1.36 (-1.85, 4.56) |  | 0.93 (-2.41, 4.26) |  | 2.26 (-0.57, 5.10) |  | 1.25 (-1.79, 4.29) |
| **Combined, late** | 1,296 |  | 1,087 |  | 411 |  | 346 |  |
| Stroke |  | -0.13 (-1.20, 0.94) |  | -1.21 (-3.11, 0.68) |  | -0.13 (-1.14, 0.88) |  | -2.17 (-4.33, 0.00) |
| Haemorrhagic stroke |  | -0.46 (-3.63, 2.70) |  | -1.97 (-3.81, -0.13)* |  | -3.45 (-4.65, -2.24)* |  | -6.10 (-11.72, -0.48)* |
| **Active ingredient and timing** | 43,715 |  | 34,288 |  | 43,715 |  | 34,288 |  |
| **Never use** | 35,716 | 0 (Reference) | 26,905 | 0 (Reference) | 35,716 | 0 (Reference) | 26,905 | 0 (Reference) |
| **Oestradiol, early** | 5,260 |  | 4,917 |  | 6,251 |  | 5,807 |  |
| Stroke |  | 0.53 (-0.62, 1.68) |  | -0.20 (-1.69, 1.30) |  | 0.55 (-0.57, 1.67) |  | -0.45 (-1.75, 0.86) |
| Haemorrhagic stroke |  | 1.22 (-2.24, 4.67) |  | 0.59 (-2.79, 3.98) |  | 2.10 (-1.81, 6.01) |  | 0.96 (-1.98, 3.91) |
| **Oestradiol, late** | 1,715 |  | 1,473 |  | 724 |  | 583 |  |
| Stroke |  | 0.25 (-0.84, 1.33) |  | -0.62 (-4.01, 2.77) |  | 0.17 (-0.82, 1.17) |  | 0.06 (-4.02, 4.14) |
| Haemorrhagic stroke |  | 0.97 (-1.31, 3.26) |  | -0.55 (-7.40, 6.30) |  | -0.65 (-7.19, 5.90) |  | -2.21 (-8.18, 3.76) |
| **CEEs, early** | 743 |  | 718 |  | 906 |  | 878 |  |
| Stroke |  | 6.94 (2.43, 11.44)* |  | 6.01 (1.33, 10.68)* |  | 4.76 (1.33, 8.19)* |  | 3.61 (-1.54, 8.76) |
| Haemorrhagic stroke |  | 2.50 (-0.98, 5.97) |  | 0.39 (-3.69, 4.48) |  | 1.94 (-2.59, 6.46) |  | 0.58 (-3.43, 4.59) |
| **CEEs, late** | 281 |  | 275 |  | 118 |  | 115 |  |
| Stroke |  | -0.15 (-2.00, 1.71) |  | -1.28 (-6.34, 3.77) |  | 0.43 (-1.31, 2.17) |  | 0.28 (-1.52, 2.08) |
| Haemorrhagic stroke |  | -0.62 (-4.65, 3.40) |  | -1.18 (-5.79, 3.44) |  | 1.84 (-1.02, 4.70) |  | -0.19 (-4.81, 4.42) |
| **Active ingredient, type and timing** | 43,715 |  | 34,288 |  | 43,715 |  | 34,288 |  |
| **Never use** | 35,716 | 0 (Reference) | 26,905 | 0 (Reference) | 35,716 | 0 (Reference) | 26,905 | 0 (Reference) |
| **Oestradiol, single, early** | 1,369 |  | 1,218 |  | 1,736 |  | 1,513 |  |
| Stroke |  | 0.65 (-1.55, 2.85) |  | 0.57 (-2.30, 3.44) |  | 1.05 (-1.12, 3.22) |  | 0.19 (-2.30, 2.68) |
| Haemorrhagic stroke |  | 0.09 (-2.24, 2.41) |  | -0.82 (-4.66, 3.01) |  | 1.21 (-4.37, 6.80) |  | -0.05 (-5.95, 5.85) |
| **Oestradiol, single, late** | 774 |  | 609 |  | 407 |  | 314 |  |
| Stroke |  | 2.02 (-0.05, 4.09) |  | 0.60 (-1.94, 3.13) |  | 2.01 (-0.24, 4.27) |  | 0.85 (-1.63, 3.33) |
| Haemorrhagic stroke |  | 6.27 (-1.91, 14.45) |  | 6.45 (-4.29, 17.19) |  | 4.11 (-14.10, 22.33) |  | 6.69 (-6.07, 19.45) |
| **Oestradiol, combined, early** | 3,891 |  | 3,699 |  | 4,515 |  | 4,294 |  |
| Stroke |  | 0.24 (-1.03, 1.51) |  | -0.36 (-1.96, 1.23) |  | 0.40 (-1.20, 1.99) |  | -0.61 (-2.06, 0.84) |
| Haemorrhagic stroke |  | 2.12 (-2.31, 6.54) |  | 1.28 (-2.35, 4.91) |  | 2.26 (-2.95, 7.48) |  | 1.51 (-2.97, 5.99) |
| **Oestradiol, combined, late** | 941 |  | 864 |  | 317 |  | 269 |  |
| Stroke |  | -0.14 (-1.47, 1.19) |  | -1.32 (-3.42, 0.78) |  | -0.15 (-1.32, 1.03) |  | -2.01 (-5.19, 1.18) |
| Haemorrhagic stroke |  | -0.52 (-5.66, 4.62) |  | -2.27 (-5.58, 1.04) |  | -5.87 (-10.00, -1.73)* |  | -6.95 (-10.70, -3.21)* |
| **CEEs, single, early** | 203 |  | 197 |  | 258 |  | 252 |  |
| Stroke |  | 7.01 (-3.39, 17.41) |  | 5.40 (-5.47, 16.28) |  | -1.79 (-4.34, 0.76) |  | -4.47 (-7.48, -1.47)* |
| Haemorrhagic stroke |  | -4.80 (-9.06, -0.54)* |  | -5.97 (-10.04, -1.90)* |  | -6.63 (-231.65, 218.39) |  | -7.69 (-20.03, 4.65) |
| **CEEs, single, late** | 108 |  | 107 |  | 53 |  | 52 |  |
| Stroke |  | -1.65 (-3.01, -0.29)* |  | -4.41 (-7.14, -1.68)* |  | 2.24 (-1.22, 5.70) |  | 1.59 (-41.85, 45.03) |
| Haemorrhagic stroke |  | -7.54 (-12.70, -2.39)* |  | -9.51 (-12.77, -6.24)* |  | 13.87 (12.59, 15.14)* |  | 12.71 (9.74, 15.68)* |
| **CEEs, combined, early** | 540 |  | 521 |  | 648 |  | 626 |  |
| Stroke |  | 5.86 (1.03, 10.69)* |  | 5.38 (0.54, 10.22)* |  | 6.97 (2.72, 11.23)* |  | 5.86 (1.32, 10.41)* |
| Haemorrhagic stroke |  | 6.29 (-5.12, 17.70) |  | 5.36 (-6.38, 17.11) |  | 5.75 (-3.76, 15.26) |  | 4.67 (-4.96, 14.31) |
| **CEEs, combined, late** | 173 |  | 168 |  | 65 |  | 63 |  |
| Stroke |  | 1.66 (-15.70, 19.02) |  | 0.40 (-1.52, 2.31) |  | -0.15 (-1.64, 1.33) |  | -0.68 (-3.28, 1.91) |
| Haemorrhagic stroke |  | 1.93 (-0.18, 4.03) |  | -0.28 (-3.94, 3.38) |  | -0.75 (-2.43, 0.93) |  | -1.11 (-3.99, 1.77) |
| **Route of administration and timing** | 43,878 |  | 34,950 |  | 43,878 |  | 32,877 |  |
| **Never use** | 35,716 | 0 (Reference) | 26,905 | 0 (Reference) | 35,716 | 0 (Reference) | 26,905 | 0 (Reference) |
| **Oral, early** | 4,144 |  | 4,108 |  | 4,946 |  | 4,898 |  |
| Stroke |  | 0.73 (-0.59, 2.05) |  | -0.16 (-1.88, 1.57) |  | 0.55 (-0.75, 1.86) |  | -0.54 (-1.97, 0.89) |
| Haemorrhagic stroke |  | 1.59 (-1.74, 4.92) |  | 1.09 (-2.32, 4.50) |  | 1.43 (-2.44, 5.30) |  | 0.89 (-1.62, 3.41) |
| **Oral, late** | 1,328 |  | 1,306 |  | 526 |  | 516 |  |
| Stroke |  | -0.26 (-1.42, 0.90) |  | -1.18 (-2.58, 0.22) |  | -0.01 (-0.97, 0.96) |  | -1.35 (-4.02, 1.32) |
| Haemorrhagic stroke |  | -0.82 (-6.57, 4.93) |  | -1.90 (-6.04, 2.25) |  | -1.00 (-4.23, 2.22) |  | -2.44 (-4.95, 0.06) |
| **Transdermal, early** | 707 |  | 695 |  | 875 |  | 861 |  |
| Stroke |  | 0.98 (-1.83, 3.78) |  | 0.30 (-2.53, 3.13) |  | 0.91 (-2.21, 4.02) |  | -0.22 (-3.30, 2.85) |
| Haemorrhagic stroke |  | 2.83 (-9.76, 15.43) |  | 1.68 (-11.24, 14.61) |  | 5.90 (-2.76, 14.57) |  | 4.82 (-3.96, 13.60) |
| **Transdermal, late** | 247 |  | 244 |  | 79 |  | 78 |  |
| Stroke |  | 1.93 (-2.40, 6.25) |  | 0.61 (-2.46, 3.69) |  | 2.98 (-1.42, 7.38) |  | 3.16 (-0.09, 6.42) |
| Haemorrhagic stroke |  | NA |  | NA |  | NA |  | NA |
| **Vaginal, early** | 487 |  | 482 |  | 853 |  | 841 |  |
| Stroke |  | 2.68 (0.85, 4.52)* |  | 2.37 (-0.03, 4.77) |  | 2.51 (0.36, 4.66)* |  | 1.66 (-0.57, 3.89) |
| Haemorrhagic stroke |  | -2.20 (-8.97, 4.57) |  | -2.10 (-31.63, 27.43) |  | -2.49 (-9.53, 4.56) |  | -3.90 (-7.02, -0.78)* |
| **Vaginal, late** | 1,249 |  | 1,210 |  | 883 |  | 851 |  |
| Stroke |  | 1.69 (0.54, 2.83)* |  | 1.13 (0.14, 2.13)* |  | 1.69 (0.37, 3.00)* |  | 1.18 (0.14, 2.23)* |
| Haemorrhagic stroke |  | 0.87 (-2.19, 3.93) |  | -0.02 (-9.19, 9.16) |  | 2.10 (-1.42, 5.63) |  | 0.71 (-2.94, 4.36) |
| **Duration and timing** | 57,368 |  | 45,759 |  | 57,368 |  | 45,759 |  |
| **Never use** | 35,716 | 0 (Reference) | 26,905 | 0 (Reference) | 35,716 | 0 (Reference) | 26,905 | 0 (Reference) |
| **≤5 years, early** | 9,000 |  | 8,084 |  | 11,001 |  | 9,882 |  |
| Stroke |  | 0.80 (0.11, 1.50)* |  | 0.55 (-0.41, 1.50) |  | 0.65 (-0.02, 1.32) |  | 0.18 (-0.75, 1.10) |
| Haemorrhagic stroke |  | 1.92 (-0.65, 4.49) |  | 0.85 (-1.12, 2.81) |  | 1.62 (-0.74, 3.98) |  | -0.74 (-1.11, 2.60) |
| **≤5 years, late** | 4,396 |  | 3,528 |  | 2,395 |  | 1,790 |  |
| Stroke |  | 0.99 (0.09, 1.90)* |  | 0.36 (-0.59, 1.31) |  | 1.27 (0.49, 2.05)* |  | 0.80 (-0.11, 1.70) |
| Haemorrhagic stroke |  | 0.91 (-1.19, 3.01) |  | -0.77 (-4.10, 2.56) |  | 0.85 (-1.92, 3.63) |  | -0.45 (-2.99, 2.08) |
| **>5 years, early** | 6,711 |  | 5,963 |  | 7,531 |  | 6,647 |  |
| Stroke |  | 0.75 (0.04, 1.45)* |  | 0.63 (-0.11, 1.38) |  | 0.71 (0.01, 1.41)* |  | 0.48 (-0.13, 1.09) |
| Haemorrhagic stroke |  | 1.34 (-1.93, 4.61) |  | 1.20 (-0.89, 3.29) |  | 2.17 (-0.99, 5.33) |  | 1.56 (-1.14, 4.27) |
| **>5 years, late** | 1,545 |  | 1,279 |  | 725 |  | 595 |  |
| Stroke |  | 0.32 (-0.51, 1.16) |  | -0.13 (-1.49, 1.23) |  | 0.46 (-0.51, 1.42) |  | -0.02 (-1.28, 1.24) |
| Haemorrhagic stroke |  | 2.95 (-0.56, 6.45) |  | 2.91 (-1.16, 6.97) |  | 0.78 (-4.59, 6.15) |  | 1.94 (-2.95, 6.84) |
| **^a^**Crude model was adjusted for age at baseline only (<55, 55–59, 60–64, 65–69 or ≥70 years). The adjusted models included age at baseline, level of education (primary school, high school or university), smoking status (never, former or current), body mass index (<25, 25–29 or ≥30 kg/m^2^), level of physical activity level (low, moderate or high) and age at menopause onset (41–46, 47–52 or 53–58 years). The 5^th^ and 1^st^ percentile differences with 95% confidence intervals, were calculated for stroke and haemorrhagic stroke, respectively.  Results significant at the 0.05 level are indicated with an asterisk (*).  PD: percentile difference, CI: confidence interval, HT: postmenopausal hormone therapy, CEE: conjugated equine oestrogen, NA: not applicable, due to 0 haemorrhagic stroke cases among users of transdermal hormone therapy. | | | | | | | | |
