# Supplementary material for: Postmenopausal hormone therapy and risk of stroke: A pooled analysis of data from population-based cohort studies
Source: PLoS Med. 2017 Nov 17;14(11):e1002445. doi: 10.1371/journal.pmed.1002445 (PMC5693286; doi:10.1371/journal.pmed.1002445)
Supplement: S4 Table — (DOCX) [file pmed.1002445.s008.docx]

| **S4 Table. Complete case analysis for stroke-free and haemorrhagic stroke-free periods in relation to the various categories of postmenopausal hormone therapy by timing of initiation. Adjusted for age at baseline** | | | | | | | | |
| --- | --- | --- | --- | --- | --- | --- | --- | --- |
|  | **Early and late HT initiation: 5-year cut-off** | | | | **Early and late HT initiation: 10-year cut-off** | | | |
|  | **N** | **Restricted^a^**  PD (95% CI) | **N** | **Non-restricted^a^**  PD (95% CI) | **N** | **Restricted^a^**  PD (95% CI) | **N** | **Non-restricted^a^**  PD (95% CI) |
| **Timing of HT initiation** | 49,956 |  | 62,476 |  | 49,956 |  | 62,476 |  |
| **Never use** | 26,905 | 0 (Reference) | 35,716 | 0 (Reference) | 26,905 | 0 (Reference) | 35,716 | 0 (Reference) |
| **Early initiation** | 17,101 |  | 19,571 |  | 20,054 |  | 22,983 |  |
| Stroke |  | 1.02 (0.35, 1.69) |  | 1.02 (0.51, 1.53) |  | 0.79 (0.19, 1.40) |  | 0.93 (0.42, 1.43) |
| Haemorrhagic stroke |  | 1.56 (-0.56, 3.69) |  | 2.24 (0.26, 4.22) |  | 1.54 (-0.40, 3.47) |  | 2.18 (0.53, 3.84) |
| **Late initiation** | 5,950 |  | 7,189 |  | 2,997 |  | 3,777 |  |
| Stroke |  | 0.53 (-0.35, 1.69) |  | 0.82 (0.12, 1.52) |  | 0.96 (0.17, 1.74) |  | 1.08 (0.39, 1.77) |
| Haemorrhagic stroke |  | 1.51 (-0.53, 3.55) |  | 1.79 (0.15, 3.42) |  | 1.51 (-1.07, 4.08) |  | 1.78 (-0.67, 4.23) |
| **Type and timing of HT** | 37,682 |  | 48,093 |  | 37,682 |  | 48,093 |  |
| **Never use** | 26,905 | 0 (Reference) | 35,716 | 0 (Reference) | 26,905 | 0 (Reference) | 35,716 | 0 (Reference) |
| **Oestrogen-only, early** | 2,774 |  | 3,243 |  | 3,590 |  | 4,257 |  |
| Stroke |  | 1.54 (0.17, 2.90) |  | 1.57 (0.07, 3.06) |  | 1.33 (0.23, 2.43) |  | 1.43 (0.34, 2.52) |
| Haemorrhagic stroke |  | -0.57 (-5.56, 4.41) |  | 0.04 (-2.27, 2.36) |  | -1.12 (-4.21, 1.98) |  | -0.43 (-3.11, 2.25) |
| **Oestrogen-only, late** | 1,884 |  | 2,459 |  | 1,028 |  | 1,445 |  |
| Stroke |  | 1.08 (-0.39, 2.54) |  | 1.42 (0.43, 2.41) |  | 1.27 (0.20, 2.34) |  | 1.67 -0.68, 2.65) |
| Haemorrhagic stroke |  | 1.09 (-3.24, 5.43) |  | 1.32 (-14.27, 16.91) |  | 2.55 (-0.94, 6.04) |  | 2.64 (0.88, 4.40) |
| **Combined, early** | 5,072 |  | 5,469 |  | 5,813 |  | 6,264 |  |
| Stroke |  | 1.11 (-0.31, 2.25) |  | 1.28 (0.22, 2.34) |  | 0.91 (-0.26, 2.07) |  | 1.16 (0.09, 2.22) |
| Haemorrhagic stroke |  | 1.47 (-1.76, 4.71) |  | 1.36 (-1.85, 4.56) |  | 1.93 (-0.69, 4.56) |  | 2.26 (-0.57, 5.10) |
| **Combined, late** | 1,087 |  | 1,296 |  | 346 |  | 411 |  |
| Stroke |  | -0.67 (-2.22, 0.89) |  | -0.13 (-1.20, 0.94) |  | -0.76 (-2.23, 0.70) |  | -0.13 (-1.14, 0.88) |
| Haemorrhagic stroke |  | -2.08 (-9.34, 5.17) |  | -0.46 (-3.63, 2.70) |  | -6.48 (-8.81, -4.15) |  | -3.45 (-4.65, -2.24) |
| **Active ingredient and timing** | 34,288 |  | 43,715 |  | 34,288 |  | 43,715 |  |
| **Never use** | 26,905 | 0 (Reference) | 35,716 | 0 (Reference) | 26,905 | 0 (Reference) | 35,716 | 0 (Reference) |
| **Oestradiol, early** | 4,917 |  | 5,260 |  | 5,807 |  | 6,251 |  |
| Stroke |  | 0.25 (-1.08, 1.58) |  | 0.53 (-0.62, 1.68) |  | 0.13 -1.15, 1.41) |  | 0.55 (-0.57, 1.67) |
| Haemorrhagic stroke |  | 0.64 (-2.83, 4.11) |  | 1.22 (-2.24, 4.67) |  | 1.37 (-2.56, 5.29) |  | 2.10 (-1.81, 6.01) |
| **Oestradiol, late** | 1,473 |  | 1,715 |  | 583 |  | 724 |  |
| Stroke |  | -0.08 (-1.08, 0.92) |  | 0.25 (-0.84, 1.33) |  | -0.04 (-1.06, 0.98) |  | 0.17 (-0.82, 1.17) |
| Haemorrhagic stroke |  | 0.20 (-17.72, 18.12) |  | 0.97 (-1.31, 3.26) |  | -1.47 (-4.21, 1.27) |  | -0.65 (-7.19, 5.90) |
| **CEEs, early** | 728 |  | 743 |  | 878 |  | 906 |  |
| Stroke |  | 6.23 (1.34, 11.12) |  | 6.94 (2.43, 11.44) |  | 3.95 (-0.32, 8.23) |  | 4.76 (1.33, 8.19) |
| Haemorrhagic stroke |  | 2.30 (-1.64, 6.24) |  | 2.50 (-0.98, 5.97) |  | 0.90 (-13.46, 15.25) |  | 1.94 (-2.59, 6.46) |
| **CEEs, late** | 275 |  | 281 |  | 115 |  | 118 |  |
| Stroke |  | -0.30 (-2.66, 2.06) |  | -0.15 (-2.00, 1.71) |  | 0.32 (-1.39, 2.03) |  | 0.43 (-1.31, 2.17) |
| Haemorrhagic stroke |  | -0.82 (-4.30, 2.67) |  | -0.62 (-4.65, 3.40) |  | 0.55 (-4.11, 5.20) |  | 1.84 (-1.02, 4.70) |
| **Active ingredient, type and timing** | 34,288 |  | 43,715 |  | 34,288 |  | 43,715 |  |
| **Never use** | 26,905 | 0 (Reference) | 35,716 | 0 (Reference) | 26,905 | 0 (Reference) | 35,716 | 0 (Reference) |
| **Oestradiol, single, early** | 1,218 |  | 1,369 |  | 1,513 |  | 1,736 |  |
| Stroke |  | 0.53 (-3.12, 4.18) |  | 0.65 (-1.55, 2.85) |  | 0.70 (-2.95, 4.35) |  | 1.05 (-1.12, 3.22) |
| Haemorrhagic stroke |  | -0.94 (-6.13, 4.26) |  | 0.09 (-2.24, 2.41) |  | -0.21 (-3.90, 3.49) |  | 1.21 (-4.37, 6.80) |
| **Oestradiol, single, late** | 609 |  | 774 |  | 314 |  | 407 |  |
| Stroke |  | -0.09 (-1.44, 1.42) |  | 2.02 (-0.05, 4.09) |  | -0.06 (-1.29, 1.17) |  | 2.01 (-0.24, 4.27) |
| Haemorrhagic stroke |  | 7.27 (-2.18, 16.72) |  | 6.27 (-1.91, 14.45) |  | 6.72 (-3.95, 17.40) |  | 4.11 (-14.10, 22.33) |
| **Oestradiol, combined, early** | 3,699 |  | 3,891 |  | 4,294 |  | 4,515 |  |
| Stroke |  | 0.16 (-1.35, 1.67) |  | 0.24 (-1.03, 1.51) |  | 0.11 (-1.29, 1.52) |  | 0.40 (-1.20, 1.99) |
| Haemorrhagic stroke |  | 1.89 (-2.36, 6.14) |  | 2.12 (-2.31, 6.54) |  | 1.71 (-2.38, 5.80) |  | 2.26 (-2.95, 7.48) |
| **Oestradiol, combined, late** | 864 |  | 941 |  | 269 |  | 317 |  |
| Stroke |  | -2.67 (-8.83, 3.49) |  | -0.14 (-1.47, 1.19) |  | -0.82 (-2.33, 0.68) |  | -0.15 (-1.32, 1.03) |
| Haemorrhagic stroke |  | -0.52 (-5.66, 4.62) |  | -0.52 (-5.66, 4.62) |  | -6.69 (-10.26, -3.13) |  | -5.87 (-10.00, -1.73) |
| **CEEs, single, early** | 197 |  | 203 |  | 252 |  | 258 |  |
| Stroke |  | 5.97 (-5.65, 17.58) |  | 7.01 (-3.39, 17.41) |  | -2.05 (-4.24, 0.14) |  | -1.79 (-4.34, 0.76) |
| Haemorrhagic stroke |  | -5.24 (-9.51, -0.97) |  | -4.80 (-9.06, -0.54) |  | -6.63 (-11.88, -1.39) |  | -6.63 (-231.65, 218.39) |
| **CEEs, single, late** | 106 |  | 108 |  | 52 |  | 53 |  |
| Stroke |  | -1.90 (-3.70, -0.10) |  | -1.65 (-3.01, -0.29) |  | 1.48 (-2.44, 5.40) |  | 2.24 (-1.22, 5.70) |
| Haemorrhagic stroke |  | -8.04 (-13.20, -2.88) |  | -7.54 (-12.70, -2.39) |  | 12.19 (4.49, 19.89) |  | 13.87 (12.59, 15.14) |
| **CEEs, combined, early** | 521 |  | 540 |  | 626 |  | 648 |  |
| Stroke |  | 5.30 (0.25, 10.35) |  | 5.86 (1.03, 10.69) |  | 6.35 (1.64, 11.05) |  | 6.97 (2.72, 11.23) |
| Haemorrhagic stroke |  | 5.00 (-7.03, 17.02) |  | 6.29 (-5.12, 17.70) |  | 4.36 (-6.03, 4.74) |  | 5.75 (-3.76, 15.26) |
| **CEEs, combined, late** | 168 |  | 173 |  | 63 |  | 65 |  |
| Stroke |  | 0.55 (-15.34, 16.43) |  | 1.66 (-15.70, 19.02) |  | -0.30 (-1.31, 0.71) |  | -0.15 (-1.64, 1.33) |
| Haemorrhagic stroke |  | 0.44 (-4.38, 5.26) |  | 1.93 (-0.18, 4.03) |  | -0.97 (-2.81, 0.86) |  | -0.75 (-2.43, 0.93) |
| **Route of administration and timing** | 34,950 |  | 43,878 |  | 34,950 |  | 43,878 |  |
| **Never use** | 26,905 | 0 (Reference) | 35,716 | 0 (Reference) | 26,905 | 0 (Reference) | 35,716 | 0 (Reference) |
| **Oral, early** | 4,108 |  | 4,144 |  | 4,898 |  | 4,946 |  |
| Stroke |  | 0.54 (-0.86, 1.94) |  | 0.73 (-0.59, 2.05) |  | 0.13 (-1.39, 1.65) |  | 0.55 (-0.75, 1.86) |
| Haemorrhagic stroke |  | 1.33 (-1.80, 4.46) |  | 1.59 (-1.74, 4.92) |  | 1.07 (-2.87, 5.01) |  | 1.43 (-2.44, 5.30) |
| **Oral, late** | 1,306 |  | 1,328 |  | 516 |  | 526 |  |
| Stroke |  | -0.78 (-2.40, 0.85) |  | -0.26 (-1.42, 0.90) |  | -0.26 (-1.34, 0.82) |  | -0.01 (-0.97, 0.96) |
| Haemorrhagic stroke |  | -0.95 (-6.89, 5.00) |  | -0.82 (-6.57, 4.93) |  | -2.15 (-11.21, 6.91) |  | -1.00 (-4.23, 2.22) |
| **Transdermal, early** | 695 |  | 707 |  | 861 |  | 875 |  |
| Stroke |  | 0.80 (-3.29, 4.88) |  | 0.98 (-1.83, 3.78) |  | 0.65 (-2.83, 4.13) |  | 0.91 (-2.21, 4.02) |
| Haemorrhagic stroke |  | 2.26 (-10.87, 15.40) |  | 2.83 (-9.76, 15.43) |  | 5.42 (-3.38, 14.23) |  | 5.90 (-2.76, 14.57) |
| **Transdermal, late** | 244 |  | 247 |  | 78 |  | 79 |  |
| Stroke |  | 1.44 (-2.90, 5.79) |  | 1.93 (-2.40, 6.25) |  | 2.33 (-24.69, 29.34) |  | 2.98 (-1.42, 7.38) |
| Haemorrhagic stroke |  | NA |  | NA |  | NA |  | NA |
| **Vaginal, early** | 482 |  | 487 |  | 841 |  | 853 |  |
| Stroke |  | 2.55 (0.64, 4.47) |  | 2.68 (0.85, 4.52) |  | 1.71 (0.03, 3.39) |  | 2.51 (0.36, 4.66) |
| Haemorrhagic stroke |  | -2.35 (-9.13, 4.44) |  | -2.20 (-8.97, 4.57) |  | -2.55 (-10.36, 5.26) |  | -2.49 (-9.53, 4.56) |
| **Vaginal, late** | 1,210 |  | 1,249 |  | 851 |  | 883 |  |
| Stroke |  | 1.56 (0.47, 2.65) |  | 1.69 (0.54, 2.83) |  | 1.57 (0.25, 2.89) |  | 1.69 (0.37, 3.00) |
| Haemorrhagic stroke |  | 0.96 (-1.74, 3.67) |  | 0.87 (-2.19, 3.93) |  | 2.03 (-1.23, 5.29) |  | 2.10 (-1.42, 5.63) |
| **Duration and timing** | 45,759 |  | 57,368 |  | 45,759 |  | 57,368 |  |
| **Never use** | 26,905 | 0 (Reference) | 35,716 | 0 (Reference) | 26,905 | 0 (Reference) | 35,716 | 0 (Reference) |
| **≤5 years, early** | 8,084 |  | 9,000 |  | 9,882 |  | 11,001 |  |
| Stroke |  | 0.65 (-0.13, 1.42) |  | 0.80 (0.11, 1.50) |  | 0.39 (-0.3, 1.16) |  | 0.65 (-0.02, 1.32) |
| Haemorrhagic stroke |  | 1.01 (-1.63, 3.65) |  | 1.92 (-0.65, 4.49) |  | 1.31 (-1.27, 3.89) |  | 1.62 (-0.74, 3.98) |
| **≤5 years, late** | 3,528 |  | 4,396 |  | 1,790 |  | 2,395 |  |
| Stroke |  | 0.78 (0.02, 1.54) |  | 0.99 (0.09, 1.90) |  | 1.16 (0.45, 1.87) |  | 1.27 (0.49, 2.05) |
| Haemorrhagic stroke |  | -0.02 (-3.02, 2.98) |  | 0.91 (-1.19, 3.01) |  | -0.05 (-3.32, 3.21) |  | 0.85 (-1.92, 3.63) |
| **>5 years, early** | 5,963 |  | 6,711 |  | 6,647 |  | 7,531 |  |
| Stroke |  | 0.52 (-1.19, 1.23) |  | 0.75 (0.04, 1.45) |  | 0.48 (-0.26, 1.22) |  | 0.71 (0.01, 1.41) |
| Haemorrhagic stroke |  | 1.33 (-1.71, 4.37) |  | 1.34 (-1.93, 4.61) |  | 1.86 (-1.56, 5.28) |  | 2.17 (-0.99, 5.33) |
| **>5 years, late** | 1,279 |  | 1,545 |  | 595 |  | 725 |  |
| Stroke |  | -0.08 (-1.05, 0.90) |  | 0.32 (-0.51, 1.16) |  | 0.10 (-0.88, 1.08) |  | 0.46 (-0.51, 1.42) |
| Haemorrhagic stroke |  | 2.77 (-0.84, 6.39) |  | 2.95 (-0.56, 6.45) |  | 1.84 (-1.04, 4.72) |  | 0.78 (-4.59, 6.15) |
| **^a^**Restricted refers to analysis based on women with complete information on all covariates included in the final multivariable adjusted model for the present study; otherwise Non-restricted.  PD: percentile difference, CI: confidence interval, HT: postmenopausal hormone therapy, CEE: conjugated equine oestrogen, NA: not applicable, due to 0 haemorrhagic stroke cases among users of transdermal hormone therapy. | | | | | | | | |
