# Supplementary material for: Postmenopausal hormone therapy and risk of stroke: A pooled analysis of data from population-based cohort studies
Source: PLoS Med. 2017 Nov 17;14(11):e1002445. doi: 10.1371/journal.pmed.1002445 (PMC5693286; doi:10.1371/journal.pmed.1002445)
Supplement: S5 Table — (DOCX) [file pmed.1002445.s009.docx]

| **S5 Table. Sensitivity analysis in which women without detailed information on timing of postmenopausal hormone therapy initiation were allocated to the early initiator group based on their age at baseline.** | | | | | | | | |
| --- | --- | --- | --- | --- | --- | --- | --- | --- |
|  | **Early and late HT initiation: 5-year cut-off** | | | | **Early and late HT initiation: 10-year cut-off** | | | |
|  | **N** | **Crude^a^**  PD (95% CI) | **N** | **Adjusted^a^**  PD (95% CI) | **N** | **Crude^a^**  PD (95% CI) | **N** | **Adjusted^a^**  PD (95% CI) |
| **Timing of HT initiation** | 74,459 |  | 51,832 |  | 80,157 |  | 54,671 |  |
| **Never use** | 35,726 | 0 (Reference) | 26,905 | 0 (Reference) | 35,716 | 0 (Reference) | 26,905 | 0 (Reference) |
| **Early initiation** | 31,554 |  | 18,977 |  | 40,664 |  | 24,769 |  |
| Stroke |  | 1.23 (0.67, 1.81) |  | 1.00 (0.44, 1.57) |  | 0.99 (0.52, 1.48) |  | 0.66 (0.13, 1.19) |
| Haemorrhagic stroke |  | 2.00 (0.82, 3.18) |  | 0.92 (-0.40, 2.24) |  | 2.38 (1.30, 3.46) |  | 1.55 (0.30, 2.80) |
| **Late initiation** | 7,189 |  | 5,950 |  | 3,777 |  | 2,997 |  |
| Stroke |  | 0.84 (0.09, 1.59) |  | 0.30 (-0.34, 0.94) |  | 1.08 (0.32, 1.48) |  | 0.56 (-0.17, 1.30) |
| Haemorrhagic stroke |  | 2.30 (0.81, 3.79) |  | 1.96 (0.37, 3.55) |  | 1.80 (0.04, 3.56) |  | 1.76 (-0.07, 3.60) |
| **Type and timing of HT** | 50,824 |  | 37,853 |  | 52,558 |  | 38,239 |  |
| **Never use** | 35,716 | 0 (Reference) | 26,905 | 0 (Reference) | 33,252 | 0 (Reference) | 26,905 | 0 (Reference) |
| **Oestrogen-only, early** | 4,121 |  | 2,865 |  | 5,957 |  | 3,914 |  |
| Stroke |  | 1.79 (0.45, 3.12) |  | 1.55 (0.47, 2.62) |  | 1.41 (0.38, 2.45) |  | 1.25 (0.08, 2.43) |
| Haemorrhagic stroke |  | 2.18 (-0.43, 4.79) |  | 1.36 (-1.63, 4.35) |  | 2.36 (0.17, 4.56) |  | 1.12 (-1.45, 3.68) |
| **Oestrogen-only, late** | 2,459 |  | 1,844 |  | 1,445 |  | 1,028 |  |
| Stroke |  | 1.43 (0.44, 2.42) |  | 0.67 (-0.23, 1.57) |  | 1.67 -0.69, 2.65) |  | 0.93 (0.22, 1.64) |
| Haemorrhagic stroke |  | 2.26 (-0.28, 4.79) |  | 2.65 (-0.50, 5.80) |  | 1.77 (-1.09, 4.63) |  | 2.72 (-0.92, 6.36) |
| **Combined, early** | 7,322 |  | 5,152 |  | 9,029 |  | 6,046 |  |
| Stroke |  | 1.60 (0.36, 2.84) |  | 0.74 (-0.67, 2.16) |  | 1.30 (0.22, 2.38) |  | 0.50 (-0.85, 1.86) |
| Haemorrhagic stroke |  | 1.87 (-0.26, 4.00) |  | 0.96 (-1.48, 3.39) |  | 2.02 (0.12, 3.92) |  | 1.39 (-0.93, 3.72) |
| **Combined, late** | 1,206 |  | 1,087 |  | 411 |  | 346 |  |
| Stroke |  | -0.12 (-1.19, 0.94) |  | -0.21 (-4.24, 3.81) |  | -0.13 (-1.18, 0.92) |  | -2.16 (-4.69, 0.37) |
| Haemorrhagic stroke |  | 1.07 (-2.79, 4.93) |  | -1.97 (-3.81, -0.13) |  | -2.92 (-8.41, -2.57) |  | -4.41 (-11.69, -2.87) |
| **Active ingredient and timing** | 46,270 |  | 34,396 |  | 47,775 |  | 34,619 |  |
| **Never use** | 35,716 | 0 (Reference) | 26,905 | 0 (Reference) | 35,716 | 0 (Reference) | 26,905 | 0 (Reference) |
| **Oestradiol, early** | 7,394 |  | 5,010 |  | 9,700 |  | 6,107 |  |
| Stroke |  | 1.01 (-0.12, 2.14) |  | -0.10 (-1.58, 1.38) |  | 0.88 (-0.14, 1.90) |  | -0.21 (-1.59, 1.18) |
| Haemorrhagic stroke |  | 2.43 (0.31, 4.54) |  | 0.86 (-1.54, 3.27) |  | 2.23 (0.38, 4.07) |  | 1.33 (-0.93, 3.58) |
| **Oestradiol, late** | 1,715 |  | 1,473 |  | 724 |  | 583 |  |
| Stroke |  | 0.26 (-0.80, 1.32) |  | -0.57 (-3.94, 2.81) |  | 0.17 (-0.81, 1.16) |  | 0.05 (-4.12, 4.21) |
| Haemorrhagic stroke |  | 3.35 (-0.24, 6.94) |  | 4.01 (-0.22, 8.24) |  | 4.45 (-2.83, 6.49) |  | 4.45 (-1.92, 10.83) |
| **CEEs, early** | 1,164 |  | 733 |  | 1,517 |  | 909 |  |
| Stroke |  | 6.74 (3.03, 10.46) |  | 5.58 (1.01, 10.15) |  | 4.63 (1.53, 7.73) |  | 2.91 (-1.35, 7.17) |
| Haemorrhagic stroke |  | 7.87 (1.51, 14.23) |  | 5.29 (-2.08, 12.67) |  | 7.26 (1.75, 12.76) |  | 6.21 (-0.81, 13.24) |
| **CEEs, late** | 281 |  | 275 |  | 118 |  | 115 |  |
| Stroke |  | -0.16 (-1.99, 1.67) |  | -1.24 (-6.28, 3.79) |  | 0.43 (-1.29, 2.14) |  | 0.28 (-1.44, 2.00) |
| Haemorrhagic stroke |  | 3.79 (-5.34, 12.91) |  | 3.34 (-2.08, 12.82) |  | 0.71 (-9.31, 10.73) |  | 0.74 (-9.75, 11.24) |
| **Active ingredient, type and timing** | 46,270 |  | 34,396 |  | 47,775 |  | 34,619 |  |
| **Never use** | 35,716 | 0 (Reference) | 26,905 | 0 (Reference) | 35,716 | 0 (Reference) | 26,905 | 0 (Reference) |
| **Oestradiol, single, early** | 1,952 |  | 1,257 |  | 2,871 |  | 1,648 |  |
| Stroke |  | 1.66 (-1.05, 4.38) |  | 0.72 (-2.15, 3.60) |  | 1.37 (-0.99, 3.73) |  | 0.70 (-2.19, 3.59) |
| Haemorrhagic stroke |  | 2.04 (-1.83, 5.90) |  | -0.73 (-4.82, 3.36) |  | 2.83 (-0.53, 6.18) |  | 0.64 (-3.18, 4.46) |
| **Oestradiol, single, late** | 774 |  | 609 |  | 407 |  | 314 |  |
| Stroke |  | 2.02 (-0.04, 4.08) |  | 0.57 (-2.08, 3.22) |  | 2.02 (-0.20, 4.24) |  | 0.85 (-1.62, 3.32) |
| Haemorrhagic stroke |  | 5.48 (-0.31, 11.27) |  | 9.68 (-4.83, 3.36) |  | 4.07 (-2.37, 10.50) |  | 10.97 (-1.51, 23.46) |
| **Oestradiol, combined, early** | 5,442 |  | 3,753 |  | 6,829 |  | 4,456 |  |
| Stroke |  | 0.90 (-0.38, 2.18) |  | -0.42 (-2.03, 1.19) |  | 0.82 (-0.35, 1.99) |  | -0.55 (-2.02, 0.93) |
| Haemorrhagic stroke |  | 2.30 (-0.07, 4.67) |  | 1.28 (-1.51, 4.08) |  | 1.97 (-0.10, 4.04) |  | 1.71 (-0.88, 4.30) |
| **Oestradiol, combined, late** | 941 |  | 864 |  | 317 |  | 269 |  |
| Stroke |  | -0.13 (-1.45, 1.18) |  | -1.34 (-3.71, 1.03) |  | -0.14 (-1.30, 1.02) |  | -1.95 (-10.23, 6.34) |
| Haemorrhagic stroke |  | 1.35 (-3.13, 5.84) |  | 0.39 (-4.35, 5.12) |  | -2.08 (-8.75, 4.58) |  | -2.69 (-10.62, 5.23) |
| **CEEs, single, early** | 369 |  | 206 |  | 510 |  | 267 |  |
| Stroke |  | 6.24 (-1.48, 13.95) |  | 6.18 (-4.17, 16.54) |  | -0.25 (-7.97, 8.47) |  | -4.56 (-7.43, -1.68) |
| Haemorrhagic stroke |  | 5.95 (-3.87, 15.77) |  | 2.31 (-9.44, 14.05) |  | 5.66 (-2.86, 14.18) |  | 1.24 (-8.94, 11.42) |
| **CEEs, single, late** | 108 |  | 107 |  | 53 |  | 52 |  |
| Stroke |  | -1.57 (-2.92, -0.23) |  | -4.47 (-7.22, -1.73) |  | 2.22 (-1.20, 5.64) |  | 1.61 (-42.02, 45.25) |
| Haemorrhagic stroke |  | 7.65 (-12.15, 27.45) |  | 7.02 (-12.70, 26.74) |  | 3.80 (2.83, 15.14) |  | 4.91 (2.86, 6.95) |
| **CEEs, combined, early** | 795 |  | 527 |  | 1,007 |  | 642 |  |
| Stroke |  | 6.61 (2.44, 10.79) |  | 4.42 (-0.56, 9.39) |  | 6.18 (2.61, 9.74) |  | 4.18 (-0.38, 8.74) |
| Haemorrhagic stroke |  | 9.001 (0.20, 17.81) |  | 6.36 (-3.89, 16.60) |  | 6.48 (-0.25, 13.21) |  | 8.11 (-1.90, 18.13) |
| **CEEs, combined, late** | 173 |  | 168 |  | 65 |  | 63 |  |
| Stroke |  | 1.68 (-15.45, 18.83) |  | 0.38 (-1.91, 2.66) |  | -0.15 (-1.61, 1.31) |  | -0.87 (-3.45, 1.70) |
| Haemorrhagic stroke |  | -0.31 (-7.99, 7.36) |  | -0.60 (-8.97, 7.76) |  | -4,57 (-20.56, 11.43) |  | -5.67 (-23.70, 12.37) |
| **Route of administration and timing** | 46,588 |  | 35,009 |  | 48,000 |  | 35,125 |  |
| **Never use** | 35,716 | 0 (Reference) | 26,905 | 0 (Reference) | 35,716 | 0 (Reference) | 26,905 | 0 (Reference) |
| **Oral, early** | 6,273 |  | 4,144 |  | 7,992 |  | 5,024 |  |
| Stroke |  | 1.34 (-0.11, 2.79) |  | -0.16 (-1.93, 1.61) |  | 0.64 (-0.53, 1.80) |  | -0.49 (-1.96, 0.99) |
| Haemorrhagic stroke |  | 3.11 (0.87, 5.34) |  | 2.08 (-0.51, 4.68) |  | 2.97 (0.98, 4.95) |  | 2.57 (0.17, 4.99) |
| **Oral, late** | 1,328 |  | 1,306 |  | 526 |  | 516 |  |
| Stroke |  | -0.28 (-1.43, 0.86) |  | -1.19 (-2.58, 0.20) |  | 0 (-0.96, 0.95) |  | -1.36 (-3.82, 1.10) |
| Haemorrhagic stroke |  | 4.69 (0.33, 9.05) |  | 3.63 (-0.73, 7.99) |  | 3.10 (-2.75, 8.96) |  | 2.23 (-4.00, 8.46) |
| **Transdermal, early** | 1,212 |  | 713 |  | 1,823 |  | 900 |  |
| Stroke |  | 2.47 (-0.13, 5.07) |  | 0.31 (-2.60, 3.22) |  | 1.18 (-0.63, 2.99) |  | 0.35 (-2.72, 3.43) |
| Haemorrhagic stroke |  | 7.32 (8.28, 13.81) |  | 3.60 (-3.32, 10.51) |  | 5.63 (0.79, 10.48) |  | 4.90 (-1.47, 11.27) |
| **Transdermal, late** | 247 |  | 244 |  | 79 |  | 78 |  |
| Stroke |  | 1.93 (-2.55, 6.41) |  | 0.59 (-2.48, 3.67) |  | 3.10 (-19.70, 25.89) |  | 3.17 (0.26, 6.08) |
| Haemorrhagic stroke |  | NA |  | NA |  | NA |  | NA |
| **Vaginal, early** | 563 |  | 487 |  | 981 |  | 851 |  |
| Stroke |  | 2.68 (1.10, 4.25) |  | 2.39 (0.05, 4.74) |  | 2.49 (0.54, 4.45) |  | 1.62 (-0.51, 3.75) |
| Haemorrhagic stroke |  | 3.82 (-2.67, 10.31) |  | 2.90 (-4.09, 9,89) |  | 2.54 (-1.87, 6.94) |  | 1.16 (-2.40, 4.71) |
| **Vaginal, late** | 1,249 |  | 1,210 |  | 883 |  | 851 |  |
| Stroke |  | 1.68 (0.55, 2.82) |  | 1.11 (0.15, 2.07) |  | 1.69 (0.40, 2.97) |  | 1.16 (0.11, 2.21) |
| Haemorrhagic stroke |  | 0.82 (-2.17, 3.81) |  | -0.14 (-2.60, 2.33) |  | 0.70 (-2.71, 4.12) |  | -0.53 (-2.96, 1.90) |
| **Duration and timing** | 61,168 |  | 45,878 |  | 63,097 |  | 45,964 |  |
| **Never use** | 35,716 | 0 (Reference) | 26,905 | 0 (Reference) | 35,716 | 0 (Reference) | 26,905 | 0 (Reference) |
| **≤5 years, early** | 12,290 |  | 8,195 |  | 15,471 |  | 10,004 |  |
| Stroke |  | 0.80 (0.14, 1.47) |  | 0.62 (-0.31, 1.55) |  | 0.70 (0.08, 1.33) |  | 0.20 (-0.73, 1.14) |
| Haemorrhagic stroke |  | 1.01 (-0.60, 2.62) |  | -0.70 (-2.50, 1.09) |  | 0.91 (-0.51, 2.33) |  | -0.05 (-1.72, 1.63) |
| **≤5 years, late** | 4,396 |  | 3,528 |  | 2,395 |  | 1,790 |  |
| Stroke |  | 0.99 (0.08, 1.91) |  | 0.29 (-0.72, 1.31) |  | 1.29 (0.52, 2.07) |  | 0.81 (-0.02, 1.64) |
| Haemorrhagic stroke |  | 0.90 (-0.92, 2.73) |  | 0.89 (-1.28, 3.05) |  | 0.62 (-1.55, 2.79) |  | 0.59 (-1.99, 3.17) |
| **>5 years, early** | 7,221 |  | 5,971 |  | 8,790 |  | 6,670 |  |
| Stroke |  | 0.78 (0.10, 1.46) |  | 0.63 (-0.11, 1.38) |  | 0.55 (-0.11, 1.20) |  | 0.50 (-0.11, 1.11) |
| Haemorrhagic stroke |  | 1.89 (0.18, 3.60) |  | 1.32 (-0.58, 3.23) |  | 2.27 (0.69, 3.85) |  | 2.00 (0.16, 3.85) |
| **>5 years, late** | 1,545 |  | 1,279 |  | 725 |  | 595 |  |
| Stroke |  | 0.32 (-0.50, 1.15) |  | -0.15 (-1.52, 1.21) |  | 0.45 (-0.60, 1.50) |  | -0.02 (-1.24, 1.20) |
| Haemorrhagic stroke |  | 1.95 (-0.68, 4.58) |  | 1.76 (-1.21, 4.73) |  | -0.16 (-3.37, 3.05) |  | -0.03 (-3.60, 3.55) |
| **^a^**Crude model was adjusted for age at baseline only (<55, 55–59, 60–64, 65–69 or ≥70 years). The adjusted models included age at baseline, level of education (primary school, high school or university), smoking status (never, former or current), body mass index (<25, 25–29 or ≥30 kg/m^2^), level of physical activity level (low, moderate or high) and age at menopause onset (41–46, 47–52 or 53–58 years). The 5^th^ and 1^st^ percentile differences with 95% confidence intervals, were calculated for stroke and haemorrhagic stroke, respectively.  PD: percentile difference, CI: confidence interval, HT: postmenopausal hormone therapy, CEE: conjugated equine oestrogen, NA: not applicable, due to 0 haemorrhagic stroke cases among users of transdermal hormone therapy. | | | | | | | | |
