# Supplementary material for: Postmenopausal hormone therapy and risk of stroke: A pooled analysis of data from population-based cohort studies
Source: PLoS Med. 2017 Nov 17;14(11):e1002445. doi: 10.1371/journal.pmed.1002445 (PMC5693286; doi:10.1371/journal.pmed.1002445)
Supplement: S6 Table — Crude and multivariable-adjusted 0.5 percentile differences are shown. Ever use was restricted to incident use. (DOCX) [file pmed.1002445.s010.docx]

| S6 **Table. Stroke-free periods in relation to the various categories of postmenopausal hormone therapy. Crude and multivariable-adjusted 0.5 percentile differences are shown. Ever use was restricted to incident use.^a^** | | | | |
| --- | --- | --- | --- | --- |
|  | **N** | **Crude^b^**  0.5 PD (95% CI) | **N** | **Adjusted^b^**  0.5 PD (95% CI) |
| **Incident use versus never use** | 39,578 |  | 29,097 |  |
| Never use | 35,716 | 0 (Reference) | 26,905 | 0 (Reference) |
| Incident use | 3,862 | 1.02 (0.27, 1.77)* | 2,192 | 1.16 (-1.44, 3.75) |
| **Timing of HT initiation using a 5-year cut-off** | 38,253 |  | 29,097 |  |
| Never use | 35,716 | 0 (Reference) | 26,905 | 0 (Reference) |
| Early initiation | 1,250 | 2.51 (-0.22, 5.24) | 1,148 | 3.32 (1.11, 5.54)* |
| Late initiation | 1,287 | 1.11 (-0.12, 2.34) | 1,044 | 0.62 (-1.33, 2.58) |
| **Type of HT** | 38,185 |  | 28,285 |  |
| Never use | 35,716 | 0 (Reference) | 26,905 | 0 (Reference) |
| Oestrogen-only HT | 1,185 | 1.13 (0.44, 1.83)* | 644 | 2.07 (-1.83, 5.98) |
| Combined HT | 1,284 | 2.24 (-0.64, 5.11) | 736 | 3.33 (1.48, 5.18)* |
| **Active ingredient** | 37,628 |  | 27,924 |  |
| Never use | 35,716 | 0 (Reference) | 26,905 | 0 (Reference) |
| Oestradiol | 1,463 | 1.06 (-0.78, 2.89) | 819 | 2.88 (0.61, 5.15)* |
| CEEs | 449 | 3.48 (0.33, 6.62)* | 200 | 2.87 (2.22, 3.52)* |
| **Route of administration** | 37,898 |  | 28,180 |  |
| Never use | 35,716 | 0 (Reference) | 26,905 | 0 (Reference) |
| Oral | 1,579 | 1.29 (-0.67, 3.25) | 854 | 1.48 (-0.02, 2.99) |
| Transdermal | 276 | -1.52 (-6.12, 3.06) | 150 | 3.20 (-2.72, 9.13) |
| Vaginal | 327 | 1.21 (1.02, 1.40)* | 271 | 2.69 (-7.38, 12.76) |
| ^a^Incident users were ever users who reported initiation of HT within the previous 12 months.  ^b^Crude model was adjusted for age at baseline only (<55, 55–59, 60–64, 65–69 or ≥70 years). The adjusted models included age at baseline, level of education (primary school, high school or university), smoking status (never, former or current), body mass index (<25, 25–29.9 or ≥30 kg/m^2^), level of physical activity (low, moderate or high) and age at menopause onset (41–46, 47–52 or 53–58 years).  P-values smaller than 0.05 are indicated with an asterisk (*)  PD: percentile difference, CI: confidence interval, HT: postmenopausal hormone therapy, CEE: conjugated equine oestrogen. | | | | |
