# Supplementary material for: Postmenopausal hormone therapy and risk of stroke: A pooled analysis of data from population-based cohort studies
Source: PLoS Med. 2017 Nov 17;14(11):e1002445. doi: 10.1371/journal.pmed.1002445 (PMC5693286; doi:10.1371/journal.pmed.1002445)
Supplement: S7 Table — (DOCX) [file pmed.1002445.s011.docx]

| **S7 Table. Sensitivity analysis including only one twin from each pair from the SALT cohort.** | | | | |
| --- | --- | --- | --- | --- |
|  | **Early and late HT initiation: 5-year cut-off** | | **Early and late HT initiation: 10-year cut-off** | |
|  | **N** | **Adjusted^a^**  PD (95% CI) | **N** | **Adjusted^a^**  PD (95% CI) |
| **Timing of HT initiation** | 47,570 |  | 47,570 |  |
| **Never use** | 24,866 | 0 (Reference) | 24,866 | 0 (Reference) |
| **Early initiation** | 16,878 |  | 19,765 |  |
| Stroke |  | 0.87 (0.30, 1.44) |  | 0.58 (0.03, 1.12) |
| Haemorrhagic stroke |  | 0.40 (-0.95, 1.75) |  | 0.85 (-0.39, 2.09) |
| **Late initiation** | 5,826 |  | 2,939 |  |
| Stroke |  | 0.18 (-0.41, 0.76) |  | 0.49 (-0.26, 1.24) |
| Haemorrhagic stroke |  | 1.89 (0.25, 3.53) |  | 1.43 (-0.68, 3.54) |
| **Type and timing of HT** | 35,296 |  | 35,296 |  |
| **Never use** | 24,866 | 0 (Reference) | 24,866 | 0 (Reference) |
| **Oestrogen-only, early** | 2,717 |  | 3,506 |  |
| Stroke |  | 1.41 (0.33, 2.49) |  | 1.02 (-0.30, 2.35) |
| Haemorrhagic stroke |  | 0.62 (-2.43, 3.67) |  | 0.82 (-1.88, 3.52) |
| **Oestrogen-only, late** | 1,775 |  | 986 |  |
| Stroke |  | 0.45 (-0.46, 1.36) |  | 0.75 (0.03, 1.49) |
| Haemorrhagic stroke |  | 2.48 (-0.79, 5.76) |  | 2.55 (-1.23, 6.35) |
| **Combined, early** | 4,906 |  | 5,608 |  |
| Stroke |  | 0.76 (-0,70, 2.21) |  | 0.38 (-1.04, 1.80) |
| Haemorrhagic stroke |  | 0.31 (-2.17, 2.78) |  | 0.81 (-1.55, 3.16) |
| **Combined, late** | 1,032 |  | 330 |  |
| Stroke |  | -1.44 (-3.38, 0.50) |  | -2.41 (-4.53, -0.29) |
| Haemorrhagic stroke |  | -0.68 (-4.80, 3.43) |  | -5.28 (-13.03, 2.46) |
| **Active ingredient and timing** | 31,974 |  | 31,974 |  |
| **Never use** | 24,866 | 0 (Reference) | 24,866 | 0 (Reference) |
| **Oestradiol, early** | 4,760 |  | 5,615 |  |
| Stroke |  | -0.33 (-1.71, 1.05) |  | -0.50 (-1.83, 0.83) |
| Haemorrhagic stroke |  | 0.29 (-2.14, 2.72) |  | 0.70 (-1.58, 2.98) |
| **Oestradiol, late** | 1,408 |  | 553 |  |
| Stroke |  | -1.16 (-2.51, 0.19) |  | 0.88 (-5.47, 3.72) |
| Haemorrhagic stroke |  | 3.49 (-0.79, 7.79) |  | 3.77 (-2.75, 10.29) |
| **CEEs, early** | 680 |  | 829 |  |
| Stroke |  | 5.55 (0.64, 10.46) |  | 3.06 (-2.47, 8.58) |
| Haemorrhagic stroke |  | 4.89 (-2.75, 12.52) |  | 5.52 (-1.60, 12.64) |
| **CEEs, late** | 260 |  | 111 |  |
| Stroke |  | -1.79 (-4.42, 0.85) |  | -0.73 (-7.35, 5.87) |
| Haemorrhagic stroke |  | -3.55 (-6.38, 13.47) |  | 1.06 (-9.76, 11.89) |
| **Active ingredient, type and timing** | 31,974 |  | 31,974 |  |
| **Never use** | 24,866 | 0 (Reference) | 24,866 | 0 (Reference) |
| **Oestradiol, single, early** | 1,191 |  | 1,481 |  |
| Stroke |  | 0.17 (-2.67, 3.01) |  | -0.11 (-2.86, 2.64) |
| Haemorrhagic stroke |  | -1.36 (-5.53, 2.81) |  | -0.22 (-4.07, 3.62) |
| **Oestradiol, single, late** | 589 |  | 299 |  |
| Stroke |  | 0.36 (-2.09, 2.80) |  | 0.57 (-1.87, 3.00) |
| Haemorrhagic stroke |  | 9.12 (0.51, 17.73) |  | 10.50 (-1.84, 22.84) |
| **Oestradiol, combined, early** | 3,569 |  | 4,134 |  |
| Stroke |  | -0.40 (-1.88, 1.08) |  | -0.58 (-2.00, 0.84) |
| Haemorrhagic stroke |  | 0.85 (-1.97, 3.66) |  | 1.15 (-1.47, 3.77) |
| **Oestradiol, combined, late** | 819 |  | 254 |  |
| Stroke |  | -1.58 (-4.01, 0.86) |  | -2.39 (-5.36, 0.58) |
| Haemorrhagic stroke |  | -0.17 (-4.96, 4.63) |  | -3.81 (-12.37, 4.75) |
| **CEEs, single, early** | 194 |  | 247 |  |
| Stroke |  | 5.12 (-6.15, 16.40) |  | -4.29 (-7.18, -1.40) |
| Haemorrhagic stroke |  | 1.19 (-10.83, 13.20) |  | 0.12 (-10.49, 10.73) |
| **CEEs, single, late** | 102 |  | 49 |  |
| Stroke |  | -4.23 (-6.44, -2.02) |  | 0.28 (-4.96, 5.52) |
| Haemorrhagic stroke |  | 6.87 (-13.34, 27.08) |  | 4.67 (2.53, 6.82) |
| **CEEs, combined, early** | 486 |  | 582 |  |
| Stroke |  | 4.93 (-0.25, 10.10) |  | 5.48 (0.71, 10.25) |
| Haemorrhagic stroke |  | 6.03 (-4.29, 16.36) |  | 7.80 (-2.30, 17.90) |
| **CEEs, combined, late** | 158 |  | 62 |  |
| Stroke |  | 0.33 (-1.73, 2.38) |  | -0.89 (-3.48, 1.70) |
| Haemorrhagic stroke |  | -0.20 (-9.07, 8.67) |  | -5.24 (-23.16, 12.68) |
| **Route of administration and timing** | 32,564 |  | 32,564 |  |
| **Never use** | 24,866 | 0 (Reference) | 24,866 | 0 (Reference) |
| **Oral, early** | 3,935 |  | 4,687 |  |
| Stroke |  | -0.32 (-1.80, 1.15) |  | -0.71 (-2.20, 0.78) |
| Haemorrhagic stroke |  | 1.75 (-0.88, 4.39) |  | 2.25 (-0.24, 4.74) |
| **Oral, late** | 1,248 |  | 496 |  |
| Stroke |  | -1.40 (-2.57, -0.24) |  | -1.72 (-4.80, 1.37) |
| Haemorrhagic stroke |  | 3.30 (-1.15, 7.75) |  | 1.76 (-4.70, 8.21) |
| **Transdermal, early** | 677 |  | 839 |  |
| Stroke |  | -0.13 (-3.58, 3.32) |  | -0.51 (-3.72, 2.71) |
| Haemorrhagic stroke |  | 2.98 (-3.96, 9.93) |  | 4.68 (-2.08, 11.43) |
| **Transdermal, late** | 236 |  | 74 |  |
| Stroke |  | 0.32 (-1.82, 2.45) |  | 3.07 (-0.27, 6.42) |
| Haemorrhagic stroke |  | NA |  | NA |
| **Vaginal, early** | 450 |  | 785 |  |
| Stroke |  | 2.10 (-0.30, 4.50) |  | 1.41 (-1.12, 3.93) |
| Haemorrhagic stroke |  | 2.27 (-4.83, 9.37) |  | -0.57 (-3.09, 4.23) |
| **Vaginal, late** | 1,152 |  | 817 |  |
| Stroke |  | 0.82 (0.01, 1.62) |  | 0.91 (-0.07, 1.89) |
| Haemorrhagic stroke |  | -0.49 (-3.00, 2.03) |  | -0.98 (-3.58, 1.63) |
| **Duration and timing** | 43,456 |  | 43,456 |  |
| **Never use** | 24,866 | 0 (Reference) | 24,866 | 0 (Reference) |
| **≤5 years, early** | 7,988 |  | 9,684 |  |
| Stroke |  | 0.52 (-0.36, 1.40) |  | 0.07 (-0.80, 0.95) |
| Haemorrhagic stroke |  | -1.12 (-2.93, 0.70) |  | -0.53 (-2.22, 1.16) |
| **≤5 years, late** | 3,463 |  | 1,767 |  |
| Stroke |  | 0.15 (-0.72, 1.03) |  | 0.56 (-0.34, 1.45) |
| Haemorrhagic stroke |  | 0.64 (-1.56, 2.84) |  | 0.30 (-2.33, 2.92) |
| **>5 years, early** | 5,888 |  | 6,562 |  |
| Stroke |  | 0.48 (-0.28, 1.25) |  | 0.32 (-0.35, 0.99) |
| Haemorrhagic stroke |  | 0.93 (-0.99, 2.85) |  | 1.59 (-0.27, 3.45) |
| **>5 years, late** | 1,251 |  | 577 |  |
| Stroke |  | -0.39 (-1.74, 0.95) |  | -0.24 (-1.47, 1.00) |
| Haemorrhagic stroke |  | 1.43 (-1.56, 4.42) |  | -0.42 (-4.10, 3.25) |
| **^a^**The adjusted models included age at baseline (<55, 55–59, 60–64, 65–69 or ≥70 years), level of education (primary school, high school or university), smoking status (never, former or current), body mass index (<25, 25–29 or ≥30 kg/m^2^), level of physical activity level (low, moderate or high) and age at menopause onset (41–46, 47–52 or 53–58 years). The 5^th^ and 1^st^ percentile differences with 95% confidence intervals, were calculated for stroke and haemorrhagic stroke, respectively.  PD: percentile difference, CI: confidence interval, HT: postmenopausal hormone therapy, CEE: conjugated equine oestrogen, NA: not applicable, due to 0 haemorrhagic stroke cases among users of transdermal hormone therapy. | | | | |
