# Supplementary material for: Postmenopausal hormone therapy and risk of stroke: A pooled analysis of data from population-based cohort studies
Source: PLoS Med. 2017 Nov 17;14(11):e1002445. doi: 10.1371/journal.pmed.1002445 (PMC5693286; doi:10.1371/journal.pmed.1002445)
Supplement: S2 Text — (DOCX) [file pmed.1002445.s013.docx]

Analysis plan: HT – STROKE associations based on COMPREHEND

Final version July 29^th^, 2016

Modified: August 25^th^, 2017

Aim of the study

The general aim is to assess the association between use of postmenopausal hormone therapy (HT) and risk of stroke.

Specifically, the aim is to assess whether early and late timing of HT initiation, respectively, in relation to menopause onset, associates with risk of stroke. Early and late HT initiation will be defined as ≤5 and >5 years since menopause onset or, alternatively, using a 10-year cut-off. Timing of HT will also be analyzed as a continuous variable.

In addition, the aim is to assess whether the potential association between HT and stroke risk varies with type of HT (single; combined), type of active ingredient (estradiol; conjugated), route of administration (oral, transdermal or local) and length of duration (>=5; <5 yrs.).

Analysis plan

1. Data cleaning, variable harmonization and classifications.
2. Variables for potential confounding control, selected on the basis of previous reports:

Age at baseline categories, age at menopause onset categories, educational level (compulsory school, high school or university), physical activity (inactive, moderate, active), alcohol consumption (never, moderate or heavy), BMI (normal, pre-obese or obese), smoking status (never, former or current), oral contraceptives (yes/no), type of menopause(surgical or natural), hypertension (yes/no), dyslipidemia (yes/no), diabetes (yes/no), family history of cardiovascular disease (CVD) (yes/no), parity categories (0-1, 2-3 or ≥4 children), and cohort.

1. Creation of endpoint variables.
   The endpoints chosen for analyses are: stroke, ischemic stroke and hemorrhagic stroke: identified from the National Patient Register (NPR) and the Cause of death register (CDR) using the international classification of disease (ICD) codes.

For the Outcome (follow up): stroke: I60 to I69 (ICD-10) or 430 to 434 and 436 to 438 (ICD-9/8) or 330-334 (ICD-7); ischemic/stroke: I63, I65, I66, (ICD-10) or 433, 434 (ICD-9/8) or 332, 333 (ICD-7); hemorrhagic/stroke: I60-I62 (ICD-10) or 430-432 (ICD-9/8) or 330, 331 (ICD-7). The following two endpoints will be analyzed: stroke (composite endpoint of ischemic stroke and hemorrhagic stroke) and hemorrhagic stroke.

1. Creation of follow-up variable: time in years since baseline, i.e. the interview date or date of filling the questionnaire, up to either date of event, or end of study.
2. Exclusion of prevalent CVD cases: Information extracted both from the NPR and the CDR: I20 to I25, I60 to I69 and G45 (TIA) (ICD-10); 410 to 414, 430 to 438 (ICD-9).
3. Assessment of homogeneity of cohorts.
4. Data pooling.
5. Strategy for confounding control: Use of Kaplan-Meier curves to describe stroke cumulative incidence by HT exposure categories (HT use, HT initiation in relation to menopause onset). Corresponding Kaplan-Meier curves with stratifications for the variables listed under point 2) above. By visual scans of these Kaplan-Meier curves, investigations if there are any factors that make the results substantially different over strata. If that is the case, we will look more in to the influences from these factors.
6. Calculation of percentile differences (PD) by use of Laplace regression, considering as dependent covariates the different aspects of HT use and HT initiation in relation to menopause onset (early initiation, late initiation and never use (reference)). The 5^th^ and 1^st^ PD were selected as the proportion for stroke and hemorrhagic stroke was 7.2% and 1.2%, respectively. The crude model will be adjusted only for age at baseline. For the multiple adjusted model, we will include factors among selected covariates from point 8) above with available information across the cohorts, one at a time in the linear predictor. The final multivariable adjusted model will include variables selected based on the visual scans of the K-M and the change in the Laplace regression point estimates.
7. Analysis restricting HT ever use to incident HT use (defined as the initiation of HT within 12 months before baseline) as compared to never use of HT. The same strategy for confounding control as for the main analyses.
8. For the analyses of timing of HT initiation as continuous variable, splines will be created to determine any gradual influence from timing of initiation on stroke risk.
9. Sensitivity analyses were performed for: 1) women who had missing information on detailed information on timing of HT initiation, 2) women who belong to the SALT (Screening Across the Lifespan Twin) cohort were twins were included, and 3) a complete case analyses for missing data on covariates included in the final model.
